# Chemical modification of group IV graphene analogs

**DOI:** 10.1080/14686996.2017.1422224

**Published:** 2018-01-31

**Authors:** Hideyuki Nakano, Hiroyuki Tetsuka, Michelle J. S. Spencer, Tetsuya Morishita

**Affiliations:** ^a^ TOYOTA CENTRAL R&D Labs, Inc., Nagakute, Japan; ^b^ School of Science, RMIT University, Melbourne, Australia; ^c^ CD-FMat, AIST, Tsukuba, Japan

**Keywords:** Two-dimensional materials, chemical modification, group IV elements, 40 Optical, magnetic and electronic device materials, 105 Low-Dimension (1D/2D) materials, 204 Optics/Optical applications, 301 Chemical syntheses/processing, 400 Modeling/Simulations

## Abstract

Mono-elemental two-dimensional (2D) crystals (graphene, silicene, germanene, stanene, and so on), termed 2D-Xenes, have been brought to the forefront of scientific research. The stability and electronic properties of 2D-Xenes are main challenges in developing practical devices. Therefore, in this review, we focus on 2D free-standing group-IV graphene analogs (graphene quantum dots, silicane, and germanane) and the functionalization of these sheets with organic moieties, which could be handled under ambient conditions. We highlight the present results and future opportunities, functions and applications, and novel device concepts.

## Graphical abstract

**Figure:**
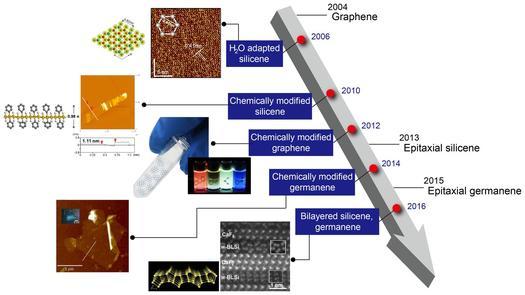

## 1. Introduction

Since the discovery of graphene by Geim and Novoselov et al. [1], a great deal of effort has been dedicated, both theoretically [2] and experimentally [3–7], to the synthesis of other similar two-dimensional (2D) materials composed of group IV elements, especially silicon (silicene) and germanium (germanene). Actually, the first report on a theoretical study of silicene was published 10 years before the discovery of the graphene [8]. Graphene has received much interest as a super-material. This is because graphene has a marvelously high electron conductivity and high mechanical strength. The only drawback to graphene is that it is gapless, which impedes its application in electronics and photonics. Therefore, many researchers have given attention to 2D silicon and/or germanium nanomaterials: targeting ‘beyond graphene’.

While graphite, having a cleavage plane, is mechanically easily exfoliated into the graphene, the structures of the other IV elements do not have cleavage planes. Therefore, these elements cannot be mechanically exfoliated into individual sheets with atomic thickness. In the history of polymer chemistry, many organic chemists have taken up the challenge to synthesize 2D silicon compounds by polymerizing silicon monomers, for example, the Wurtz reaction [9–12]. Unfortunately, these efforts never managed to achieve the desired crystalline 2D silicon material; only amorphous polyalkyl oligosilanes were obtained. In 2010, Lalmi et al. succeeded in fabricating silicene on an Ag substrate under high vacuum conditions [13]. Thereafter, the experimental study of silicene-related materials received an explosion of interest (see following references; monolayer silicene on Ag substrate [14–19], multilayer silicene on Ag substrate [20–25] or other substrate [26–28], chemical modification of silicene [29,30]). Unconventional allotropes of Si, prepared under high-vacuum conditions, can be an alternative option to keep up with the current technology mainstream, and among them, silicene can be a promising option enabling the use of dimensionally reduced Si. In 2015, the first silicene transistor was synthesized through a complex process; however, the performance was modest and the lifetime of the transistor was too short [31]. This was because the silicene deposited under high-vacuum conditions was comprised almost entirely of *sp* ^2^ silicon, and was easily oxidized under ambient conditions. Therefore, free-standing group IV elemental sheets having high stability under ambient conditions is a challenging issue. These challenges include: addressing the gapless nature of graphene and increasing the stability of silicene and germanene under ambient conditions. In this review, to compensate for the weaknesses of mono-elemental classes of 2D-Xenes (X=C (graphene), Si (silicene), Ge (germanene)), we will focus on 2D free-standing group IV graphene analogs (graphene quantum dots, silicane, and germanane). In these materials, every atom in the framework features a covalently bound ligand, thereby removing π-bonding. These ligands directly couple to the half-filled *p*_*z*_ orbitals to produce a gapped, semiconducting band structure; and the magnitude of this bandgap depends on both the framework element and the identity of the covalent ligand.

## 2. Graphene quantum dots

**Figure 1.**
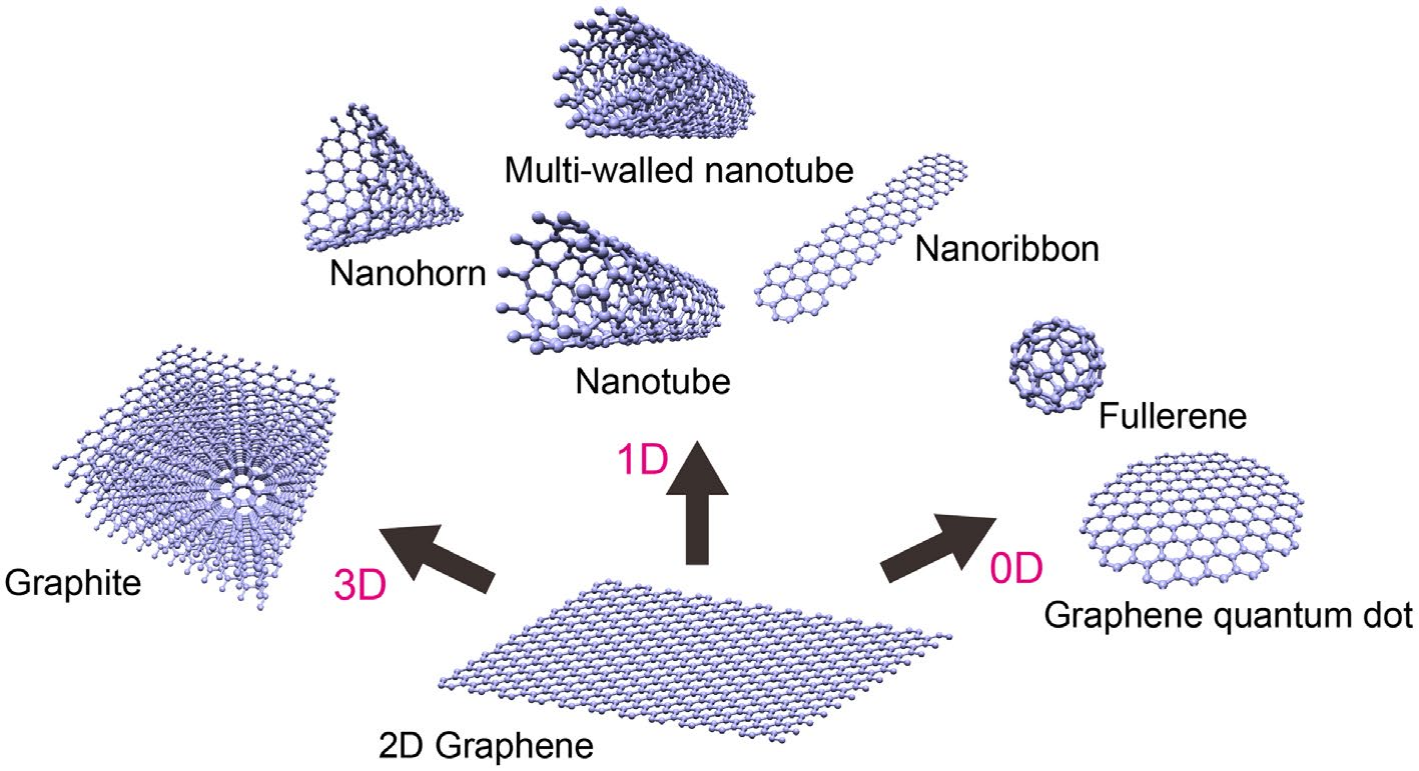
Classification of carbon allotropes derived from graphene.

Carbon, as the main building block of organic compounds, is a fundamentally important element for all living organisms. The unique ability of carbon atoms to participate in strong covalent bonds with other carbon atoms in various hybridization states (*sp*, *sp* ^*2*^, *sp* ^*3*^) enables them to form very diverse structures. Among the diverse carbon allotropes, graphene is a perfect construction of *sp* ^*2*^ carbon atoms arranged in a planar honeycomb lattice [1]. Graphene is the fundamental building block for constructing many other 0D, 1D, and 3D allotropes: fullerenes, single or multi-walled nanotubes, nanohorns, nanoribbons, graphene quantum dots, and graphite (Figure 1). Since its discovery in 2004, by Geim and Novoselov et al. using sticky tape to peel atomically the thin layers of graphene from kish graphite, it has attracted intense scientific interest because of its extraordinary properties including high carrier mobility, transparency, mechanical strength, and chemical stability. Its combination of attractive characteristics suggests that it can potentially replace silicon in many applications [32–36]. However, an important shortcoming of graphene is its gapless nature, which impedes its application in electronics and photonics.

Among these graphene derivatives, graphene quantum dots (GQDs), nanometer-sized fragments of graphene, have been recently implemented in bandgap applications because their strong quantum-confinement and edge effects produce exceptional photoluminescence properties [37–41]. Although graphene exhibits an infinite exciton Bohr radius, quantum-confinement can occur in graphenes of any finite size. That confinement is expected to result in many interesting phenomena that cannot be observed in conventional semiconductor nanocrystals. It is also possible to synthesize GQDs through solution chemistry to obtain well-defined structures with atomic precision unachievable for any other semiconductor materials. Consequently, luminescent colloidal GQDs spanning infrared, visible, and blue spectral ranges have been anticipated to find optoelectronic applications.

**Figure 2.**
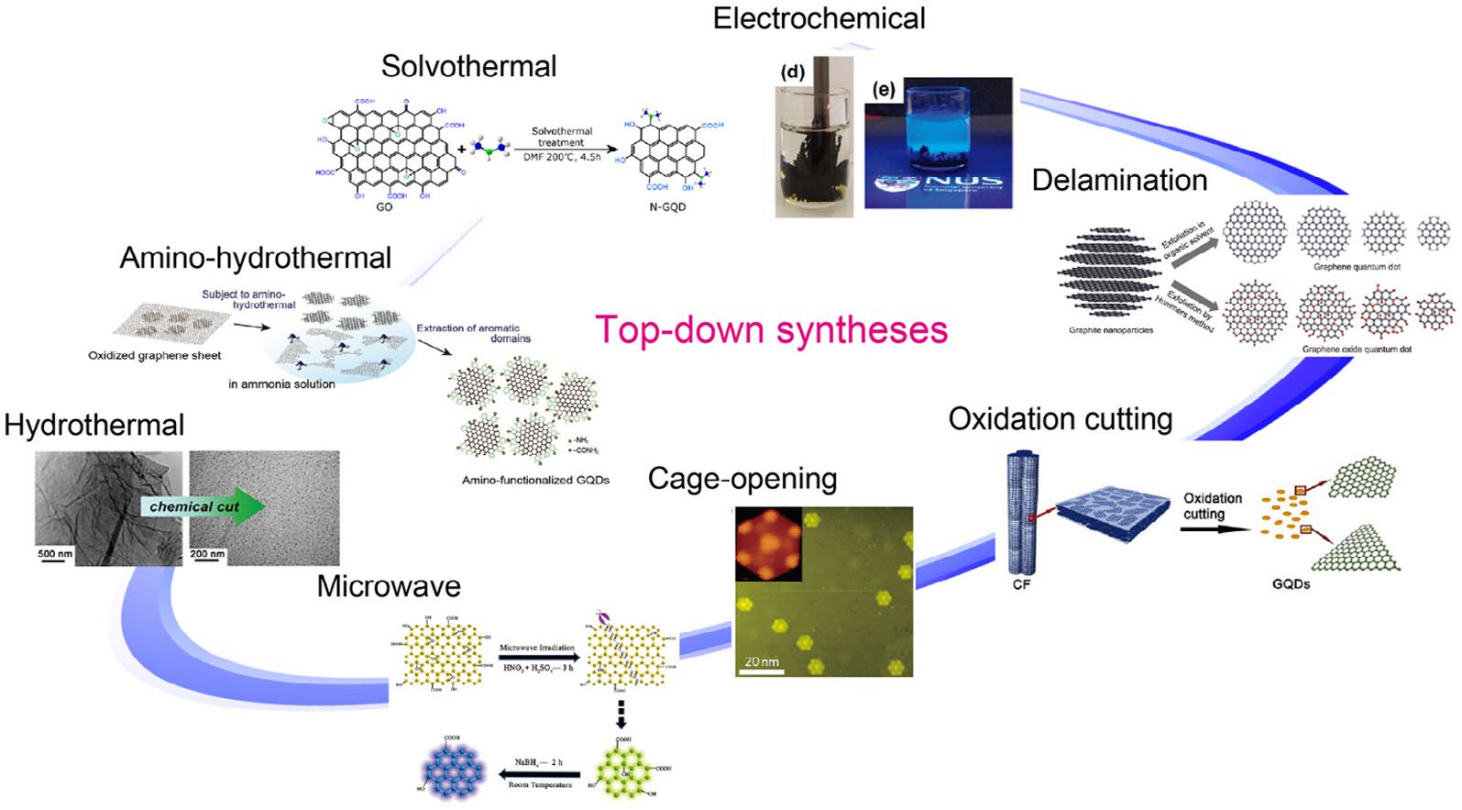
Methodologies for synthesizing GQDs through top-down routes. Reproduced with permission from [42–49]. Copyright 2010, 2012, 2013, Wiley-VCH. Copyright 2009, 2012, 2013, American Chemical Society. Copyright 2011, Macmillan Publishers Limited.

Two main approaches have been developed to synthesize GQDs: i) cutting graphene sheets through top-down routes, and ii) building up small precursor aromatic molecules via bottom-up routes. Raw materials for preparing GQDs through top-down routes are so abundant that GQDs can be prepared on a large scale. Consequently, top-down methods are an obvious choice for preparing monolayer GQDs. Various top-down methods are presented in Figure 2. Top-down methods involve the breaking down of large oxidized graphene sheets, carbon fibers, fullerenes, graphite nanoparticles, or graphite rods into small pieces of graphene sheets using hydrothermal [42,43] or solvothermal exfoliation [44], electrochemical oxidation [45,46], acidic oxidation [47,48], and microwave radiation methods [49]. However, for bottom-up methods, GQDs can be typically realized by a dehydrogenation reaction of small organic precursor molecules [50–52]. Nevertheless, it is difficult to obtain a monolayer graphene quantum dot.

The GQDs synthesized via top-down methods usually contain negatively charged and hydrophilic oxygenated groups, making them soluble in polar solvents such as water, dimethylformamide (DMF), and ethanol. Sizes of GQDs obtained from different preparation methods differ, and mostly range from 3 to 20 nm. GQDs are defined as one or more layers of graphene sheets less than 5 nm in thickness [43]. Most synthesized GQDs are circular or elliptical in shape, but hexagonal and quadrilateral GQDs have also been obtained.

### 2.1. Optical properties

The presence of finite-sized molecular *sp* ^*2*^ domains in GQDs and CO-related quasi-molecular fluorophores, induced by the adsorption of oxygen functional groups, can confine π electrons and consequently give off photoluminescence (PL) that is dictated by the nature of the *sp* ^*2*^ domains and surface functional groups. GQDs synthesized by top-down approaches typically exhibit blue PL, centered at 420–450 nm, that is ascribed mainly to the limited tunability of the sizes and shapes of the *sp* ^*2*^ domains and the adsorption of oxygen functional groups [42]. GQDs with tunable PL can be synthesized through the precise control of *sp* ^*2*^ domain sizes, edge structures, and surface functional groups.

**Figure 3.**
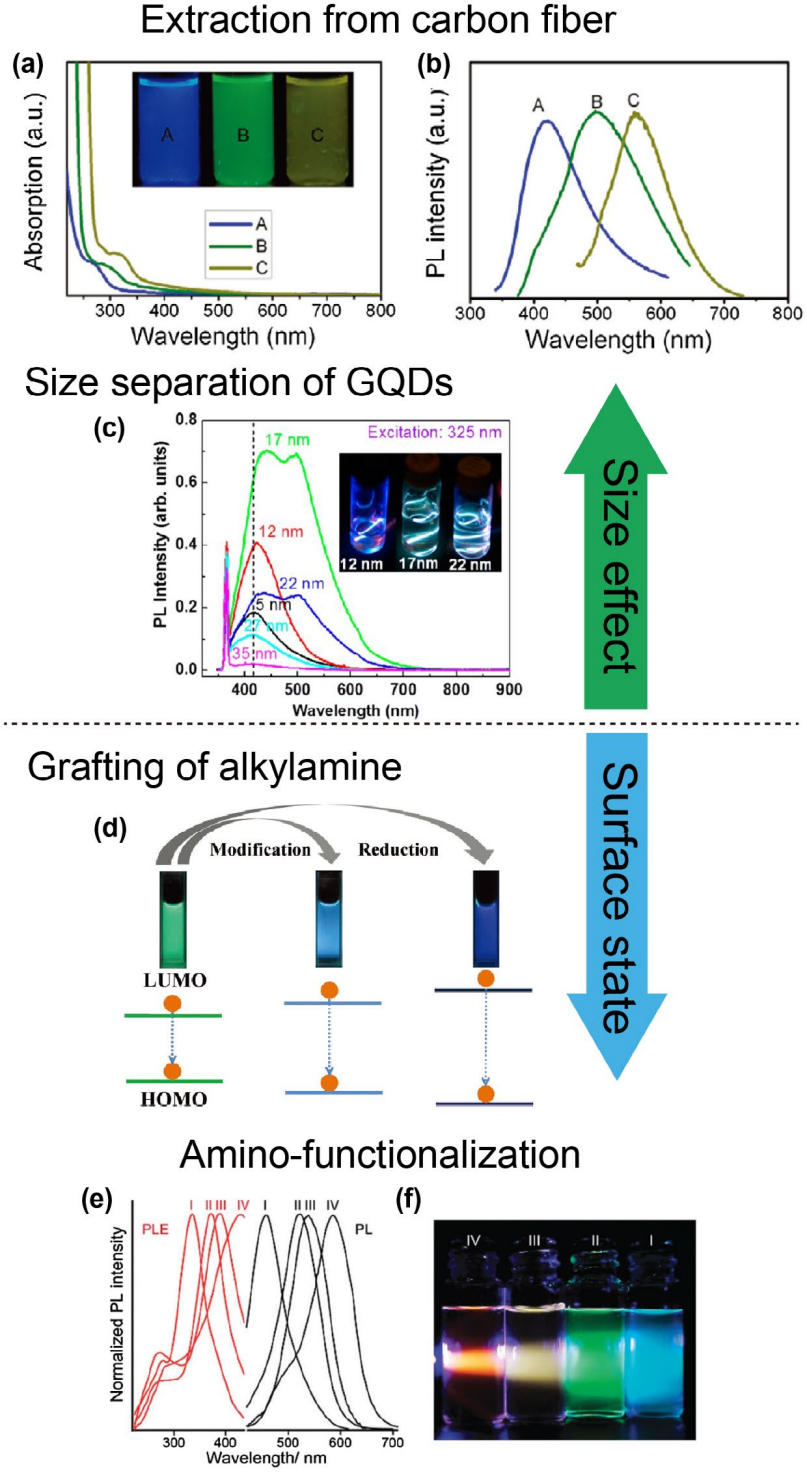
Changes in the optical properties of GQDs-based on size and surface state. (a) UV–vis spectra of GQDs A, B, and C, correspond to synthesis reaction temperatures of 120, 100, and 80 °C, respectively. (b) PL spectra of GQDs with different emission color. Reproduced with permission from [47]. Copyright 2012, American Chemical Society. (c) Size-dependent PL spectra excited at 325 nm for 5–35 nm GQDs in deionized (DI) water. Inset: different colors of luminescence from GQDs depending on their average size for three typical GQDs of 12, 17, and 22 nm. Reproduced with permission from [56]. Copyright 2012, American Chemical Society. (d) Bandgap changes for GQDs, m-GQDs, and r-GQDs. Reproduced with permission from [59]. Copyright 2012, Wiley-VCH. (e) Normalized PL (black lines) and PL excitation (red lines) spectra from aqueous amino-functionalized GQDs (afGQDs) solutions prepared under different conditions. (f) PL image of an aqueous afGQDs solution under excitation from a blue light-emitting diode (LED) (410 nm). Reproduced with permission from [57]. Copyright 2015, Royal Society of Chemistry.

Eda et al. reported the presence of isolated *sp* ^*2*^ clusters within the carbon–oxygen *sp* ^*3*^ matrix as the origin of PL in GQDs. The energy gap was found to vary with the *sp* ^*2*^ cluster size [53]. Sk et al. investigated the PL mechanism of GQDs using density functional theory (DFT) and time-dependent DFT (TDDFT) calculations [54]. These studies revealed that the PL of zigzag-edged GQDs can be tuned to the entire visible light spectrum by varying the diameter of the GQDs. Experimentally, Peng et al. synthesized GQDs of different sizes, 2–4 nm (A in Figure 3(a) and (b)), 5–7 nm (B in Figure 3(a) and (b)), and 8–10 nm (C in Figure 3(a) and (b)), through acid treatment and chemical exfoliation of pitch carbon fibers. These GQDs emitted different PL, changing from blue/green to yellow [47]. Ye et al. reported that GQDs prepared via acidic oxidation from coal showed a PL transition from blue to yellow as their size increased from 2.3 to 5.8 nm [55]. In addition, Kim et al. showed that the peak energy of the PL spectra decreases with as the average GQD size changes from 5 to 35 nm (Figure 3(c)) [56]. Nevertheless, as has been observed for absorption spectra, size-dependent PL might not tell the whole story for GQDs.

### 2.2. Chemical modifications

Chemical functionalization is an effective means of tailoring the electronic characteristics of GQDs, especially because of the remarkable quantum-confinement and edge effects of quantum dot-sized graphene. Various chemical functional groups, such as amines [57,58], alkylamines [59], and thiols [60], have been attached to GQDs. Chemical modifications using molecules with strong electron-donating or electron-accepting abilities cause a marked PL wavelength shift because of the marked changes to the electronic characteristics of the GQDs. Our group initially demonstrated that the emission wavelengths of GQDs can be widely tuned (blue to orange) by controlling the degree of amine functionalization (Figure 3(e) and (f)) [42,57]. Zhu et al. also reported that green PL GQDs become blue after replacing carboxyl functional groups with alkylamine functional groups (Figure 3(d)) [59]. Both experimental results and DFT calculations proved the band gap decrease via tuning of the concentration of attached alkylamine groups. Reduction or oxidation of surface oxygen groups might affect the optical properties completely, resulting in different visible light [61].

**Figure 4.**
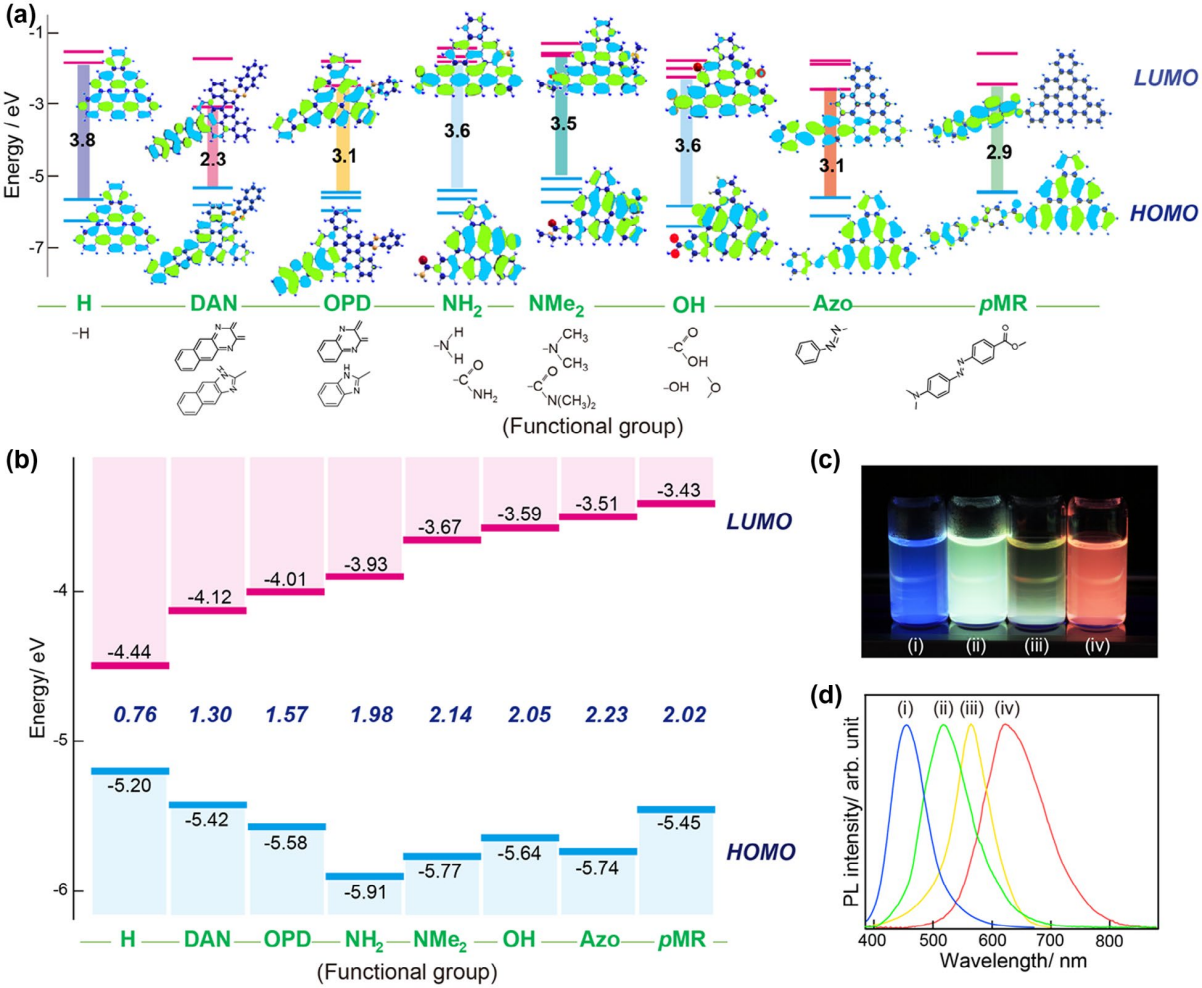
Modulation of the optical properties in GQDs through chemical functionalization alone. Energy levels and PL for nitrogen-functionalized graphene quantum dots (NGQDs). (a) Predicted energy level diagrams for graphene with different functional groups. (b) Measured energy level diagram for NGQDs. (c) PL image of NGQDs in aqueous solution excited using a UV lamp (365 nm): (i) Azo-GQDs; (ii) NH_2_-GQDs; (iii) OPD-GQDs; and (iv) DAN-GQDs. (d) Corresponding normalized PL spectra (excited at 380 nm) from aqueous dispersions of NGQDs. Reproduced with permission from [62]. Copyright 2016, Wiley-VCH.

Our group also functionalized GQDs using various chemical groups that had been selected based on *ab initio* calculations [62]. Functionalization occurred through nucleophilic substitution and dehydration with an amine group on precursors containing epoxide bridges and hydroxyls on oxidized GQDs (o-GQDs), respectively. The change in highest occupied and lowest unoccupied molecular orbitals (HOMO/LUMO) levels of the GQDs upon functionalization is depicted in Figure 4. The HOMO/LUMO energy levels changed continuously with different nitrogen-containing functional groups due to the strong orbital interactions. Each species of nitrogen-containing group altered the entire electronic structure of the nitrogen-functionalized graphene quantum dot (NGQD): those functionalized with a primary amine (NH_2_-GQDs) or dimethyl amine(NMe_2_-GQDs) had one degenerate graphene HOMO orbital raised to a higher energy level due to the strong orbital interactions, whereas NGQDs functionalized with o-phenylenediamine (OPD-GQDs), diaminonaphthalene (DAN-GQDs), azo (Azo-GQDs), or p-methyl red (*p*MR-GQDs) had the LUMO orbital shift to a lower energy level. The HOMO/LUMO energy levels can be tuned continuously by adjusting the strength of the orbital interactions using different nitrogen-containing groups and adjacent groups. Tertiary amines (NMe_2_) have stronger orbital interactions with the HOMO than primary amines (NH_2_), whereas DAN and *p*MR have stronger orbital interactions with the LUMO than OPD and Azo. Because of the push–pull effect, the existence of adjacent groups with opposite effects also enhances the energy gap narrowing. The apparent HOMO–LUMO gap varied from 1.30 to 2.23 eV. The energy gap tunability was also manifested in a distinct red shift of the PL band maxima and UV absorption edges with a narrowing of the HOMO–LUMO gap. After excitation with a UV lamp (365 nm), these NGQDs exhibited strong colorful PL from blue to red. The optical properties of GQDs are known to depend on a combination of factors: size, shape, and surface functionality. All species of the NGQDs had almost identical sizes and shapes. Therefore, the tunability of the PL was attributable to differences in the surface functionality of the NGQDs only. Table 1 summarizes the classification of substituent effects on orbital interaction and energy gap of GQDs. The electron-donating character and energy levels of substituent are key factors for narrowing energy gap of GQDs. The substituents with electron-donating character raise HOMO to a higher energy, causing narrowing of the energy gap. In addition, the nitrogen-substituted aromatics induce additional energy levels between the LUMO and HOMO, markedly narrowing the band gap. These results imply that the HOMO/LUMO levels and energy gaps can be modified for various optoelectronic device applications.

**Table 1.** Classification of substituent effects on.

Substituent	Character relative to H	Orbital interaction	Narrowing effect of energy gap
‒COOH	Electron withdrawing	LUMO	Weak
	Electron withdrawing	LUMO	Weak
‒OH	Electron donating	HOMO	Weak
	Electron donating	HOMO	Moderate
‒NH_2_	Electron donating	HOMO	Moderate
‒N(CH_3_)_2_	Electron donating	HOMO	Moderate
	Electron donating	LUMO	Moderate
	Electron donating	LUMO	Moderate
	Electron donating	LUMO	Strong
	Electron donating	LUMO	Strong

### 2.3. Applications for optoelectronic devices

**Figure 5.**
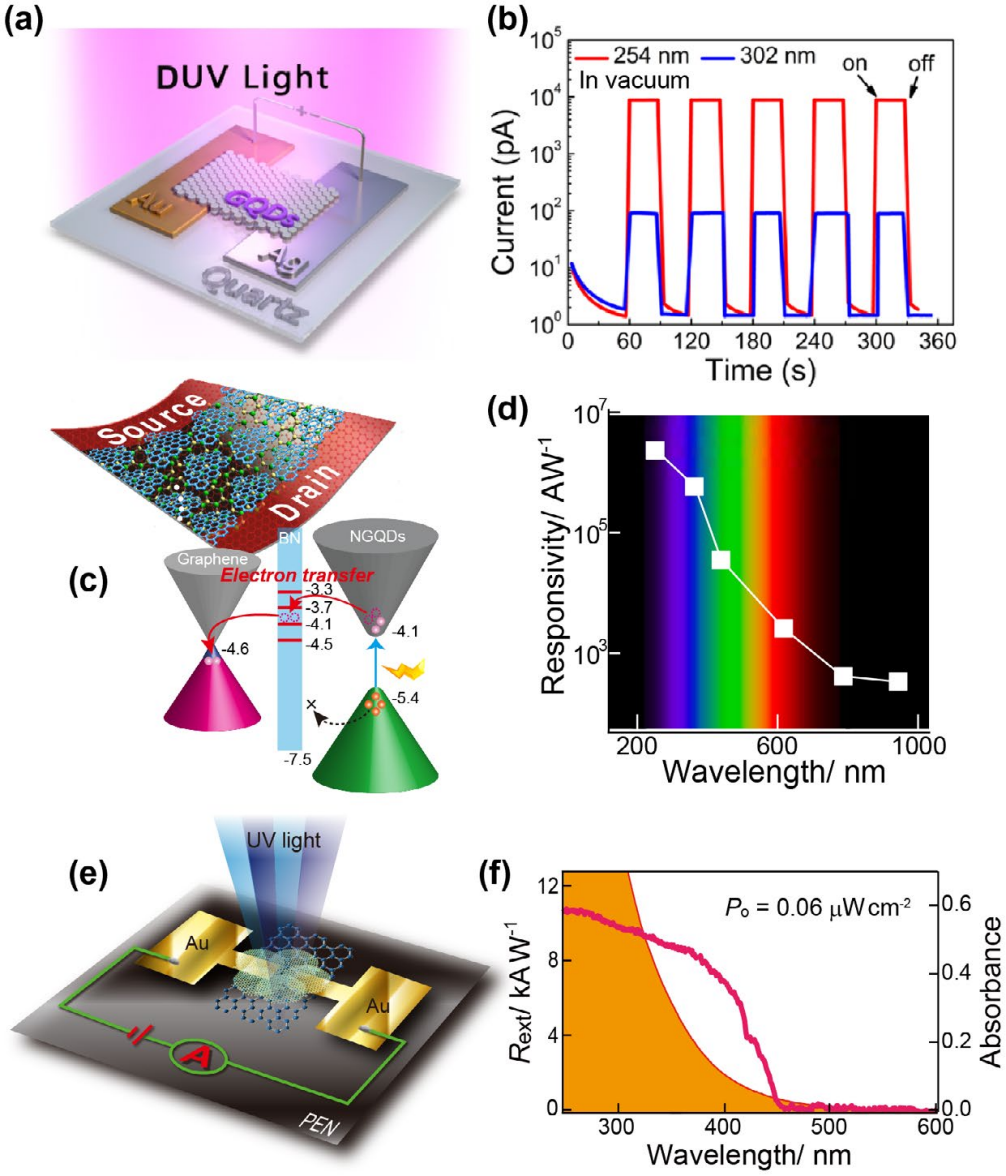
GQDs-based photodetectors. (a) Schematic illustration of the configuration for photoconductive measurements of GQDs with asymmetric electrodes. (b) Time response of the device under pulsed 254 or 302 nm DUV light (42 μW cm^−2^). Reproduced with permission from [63]. Copyright 2015, American Chemical Society. (c) Schematic of the energy level alignment for the photodoping effect. (d) Photoresponsivity vs. optical irradiation power (laser wavelengths of 254–940 nm) for the DAN-GQD/BN-NS@GFET hybrid PD. Reproduced with permission from [54]. Copyright 2016, Royal Society of Chemistry. (e) Schematic representation of the NMe_2_-GQD@GFET hybrid PD. PEN stands for polyethylene naphthalate (f) Spectral response (left axis) from the NMe_2_-GQD@GFET PD (*V*_SD_ = 0.1 V, *V*_G_ = 0 V, 0.06 μW cm^−2^). The solid orange area (right axis) represents the UV–vis absorption spectrum of a NMe_2_-GQD solution. Reproduced with permission from [64]. Copyright 2017, Macmillan Publishers Limited.

Researchers have designed and fabricated various optoelectronic devices using chemically modified GQDs. First, we will demonstrate the applications of GQDs in photodetectors. Commonly, GQDs have a strong UV absorption peak at approximately 250 nm, which is attributed to the π–π* transition. Therefore, deep-UV (DUV) photodetection has been investigated for GQD-based photodetectors (PDs). For example, Zhang et al. fabricated GQD-based DUV PDs with asymmetric electrodes combined with Au and Ag, as shown in Figure 5(a) and (b). The GQD-based DUV PD possessed a high on/off ratio, even under weak irradiation of 8 μW cm^−2^ with a response time of 64 (rise) and 43 (decay) ms [63]. Our group has also shown a GQD/graphene field-effect transistor (GFET) hybrid UV PD using NMe_2_-GQDs with a bandgap of approximately 3.3 eV (Figure 5(c) and (d)) [64]. The NMe2-GQD@GFET PD exhibits high photoresponsivity and detectivity of approximately 1.5 × 10^4^ A W^−1^ and 5.5 × 10^11^ Jones, respectively, in the DUV region as short as 255 nm. GQDs were also used for broadband PDs. Kim et al. demonstrated a broadband photodetection range, from UV to near-infrared wavelengths, using PDs consisting of multilayer GQDs sandwiched between graphene sheets [65]. However, their photoresponsivity was limited to 0.2–0.5 A W^−1^. Recently, our group also produced a high-performance broadband PD that used a hybrid consisting of graphene and NGQDs [66]. The NGQDs@GFET PD with boron nitride nanosheets (BN-NSs) buffer layers exhibited enhanced photoresponsivity and detectivity in the DUV region of approximately 2.3 × 10^6^ A W^−1^ and 5.5 × 10^13^ Jones (Figure 5(e) and (f)), respectively. The high level of photoresponsivity extended even into the near-infrared region (approximately 3.4 × 10^2^ A W^−1^ and 8.0 × 10^9^ Jones). The high photoresponsivity was a consequence of strong light absorption and long-lived photogenerated carriers in the NGQDs. In addition to strong light absorption, NGQDs have bandgap tunability that covers a range of absorption wavelengths, from DUV to the infrared region.

**Figure 6.**
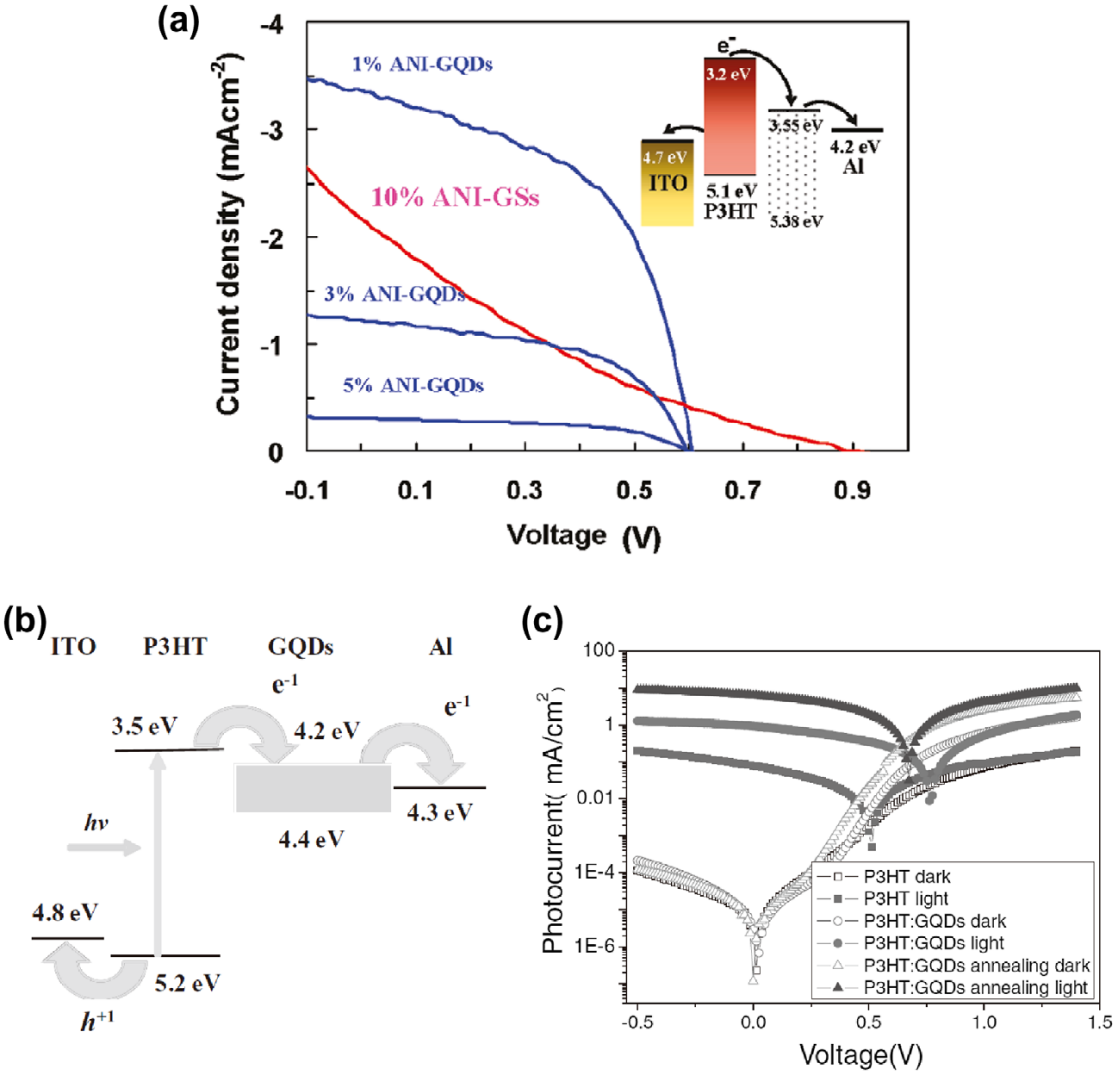
GQDs-based organic photovoltaic devices. (a) J–V characteristics of the devices based on aniline (ANI) and GQDs with different GQDs content and on aniline and graphene sheets (GSs, under optimized conditions) annealed at 160 °C for 10 min, under AM 1.5G 100 mW illumination. Reproduced with permission from [67]. Copyright 2011, American Chemical Society. (b) Schematics of the ITO/PEDOT:PSS/P3HT:GQDs/Al device containing indium tin oxide (ITO), poly(ethylene dioxythiophene)–polystyrene sulfonic acid (PEDOT:PSS) and poly(3-hexylthiophene-2,5-diyl) (P3HT). (c) J–V characteristic curves for the ITO/PEDOT:PSS/P3HT/Al, ITO/PEDOT:PSS/P3HT:GQDs/Al and ITO/PEDOT:PSS/P3HT:GQDs/Al devices after annealing at 140 °C for 10 min. Reproduced with permission from [68]. Copyright 2011, Wiley-VCH.

The broad absorption and electron transportation capabilities of GQDs facilitate the solution performance of processed organic and dye-sensitized solar cells. Gupta et al. reported that GQDs blended with poly(3-hexylthiophene-2,5-diyl) (P3HT) polymer markedly improved the photovoltaic characteristics (Figure 6(a)) [67]. The LUMO energy level of GQDs is –3.55 to 5.38 eV, which is between those of the LUMO and Al, forming an electron transport cascade. This band structure will facilitate the dissociation of excitons. In addition, P3HT/GQD blends provide uniform and fine features, suggesting nanoscale phase separation, which enhances the exciton migration to the donor–acceptor interface, resulting in a decrease in the resistance and a corresponding increase in the fill factor. Consequently, enhanced photoconversion efficiency was obtained. Li et al. fabricated a bulk heterojunction (BHJ) organic solar cell with improved performances based on a composite layer of P3HT and GQDs (Figure 6(b) and (c)) [68].

**Figure 7.**
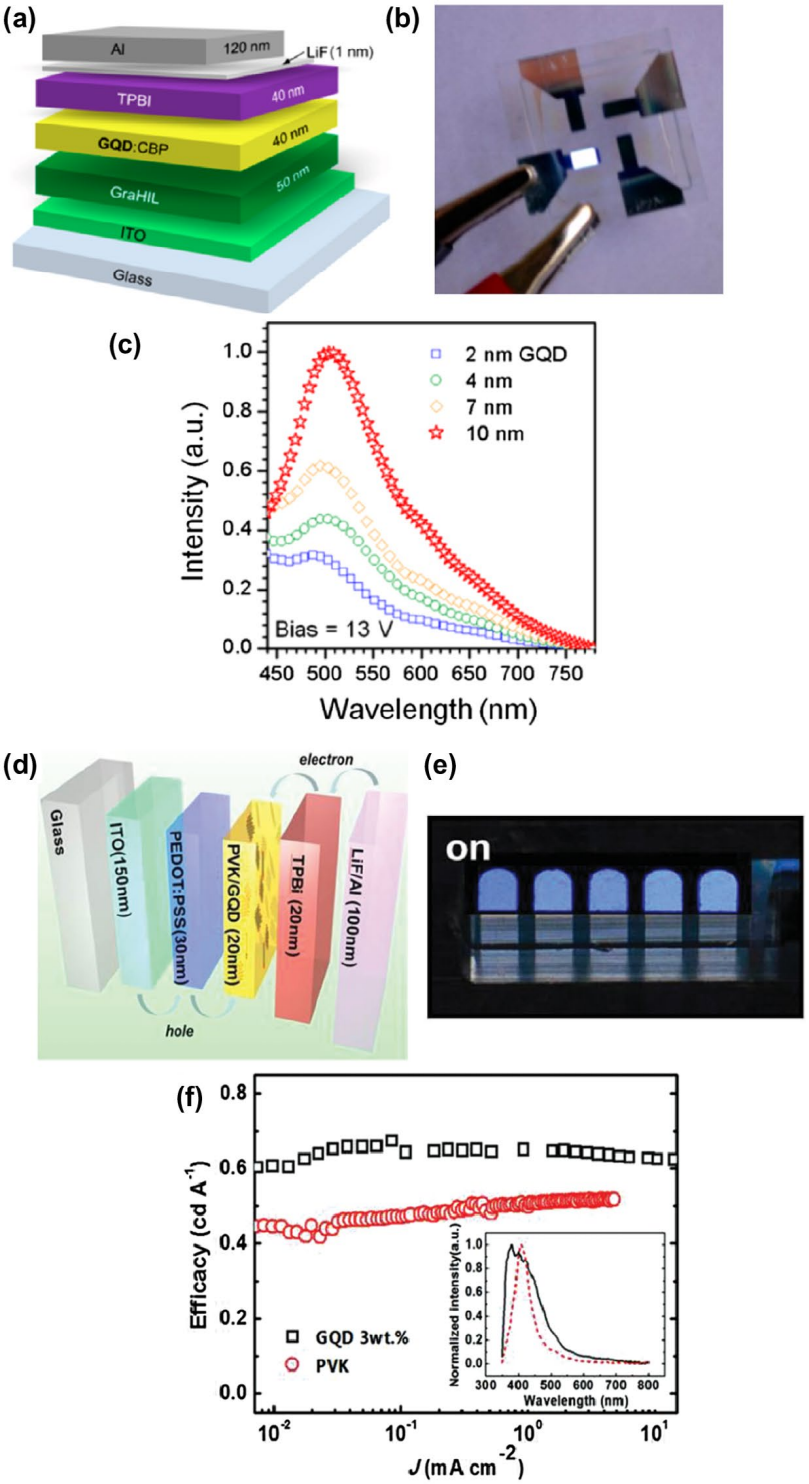
GQDs-based LEDs. (a) Schematic of organic LEDs (OLED) employing GQDs. GraHIL and TPBI (1, 3, 5-tri(phenyl-2-benzimidazolyl)-benzene) are hole and electron transporting layers, respectively. (b) Photograph of white-light emission from an OLED employing 10 nm GQDs. (c) Normalized PL intensity of a set of OLEDs at a fixed bias (13 V). Reproduced with permission from [69]. (d) Schematic illustration of the GQD-LEDs structure and the corresponding band diagram. (e) Electroluminescence image of GQD-LEDs consisting of five emitting areas. (f) Luminous efficiencies and emission spectra of the devices. Copyright 2014, American Chemical Society. Reproduced with permission from [70]. Copyright 2014, Wiley-VCH.

Finally, GQDs have been examined as luminophores in LEDs. Kwon et al. demonstrated that GQD-LEDs with alkylamine-terminated GQDs prepared by amidative cutting of tattered graphite generates white light emissions with approx. 0.1% of external quantum efficiency (EQE) (Figure 7(a)–(c)) [69]. Song et al. reported deep-blue GQD-LEDs with luminous efficiency of 0.65 cd A^−1^, using GQDs fabricated from graphite intercalation compounds [70]. Son et al. reported white LEDs with 0.18% of EQE using a zinc oxide/GQDs hybrid (Figure 7(d)–(f)) [71]. At 15 V applied bias and with optimal CIE coordinates (0.23, 0.20), the maximum brightness reached approximately 798.1 cd m^−2^. GQDs have been used as phosphors to convert blue light to white light. Our group demonstrated that GQDs/clay films convert blue LED to pure white light emissions with a CIE coordinate of (0.33, 0.37) [57]. Luk et al. applied GQDs/agar composites as a color converting material in white LEDs. Luminous efficiency of 42.2 lm W^−1^ and light conversion efficiency of 61.1% was achieved [72]. These results demonstrate the possibility of using GQDs in light-emitting devices; however, the main challenge for practical applications remains the improvement of quantum efficiency.

## 3. Monolayer silicene derivatives

### 3.1. Zintl silicides and derived layered silicon compounds

**Figure 8.**
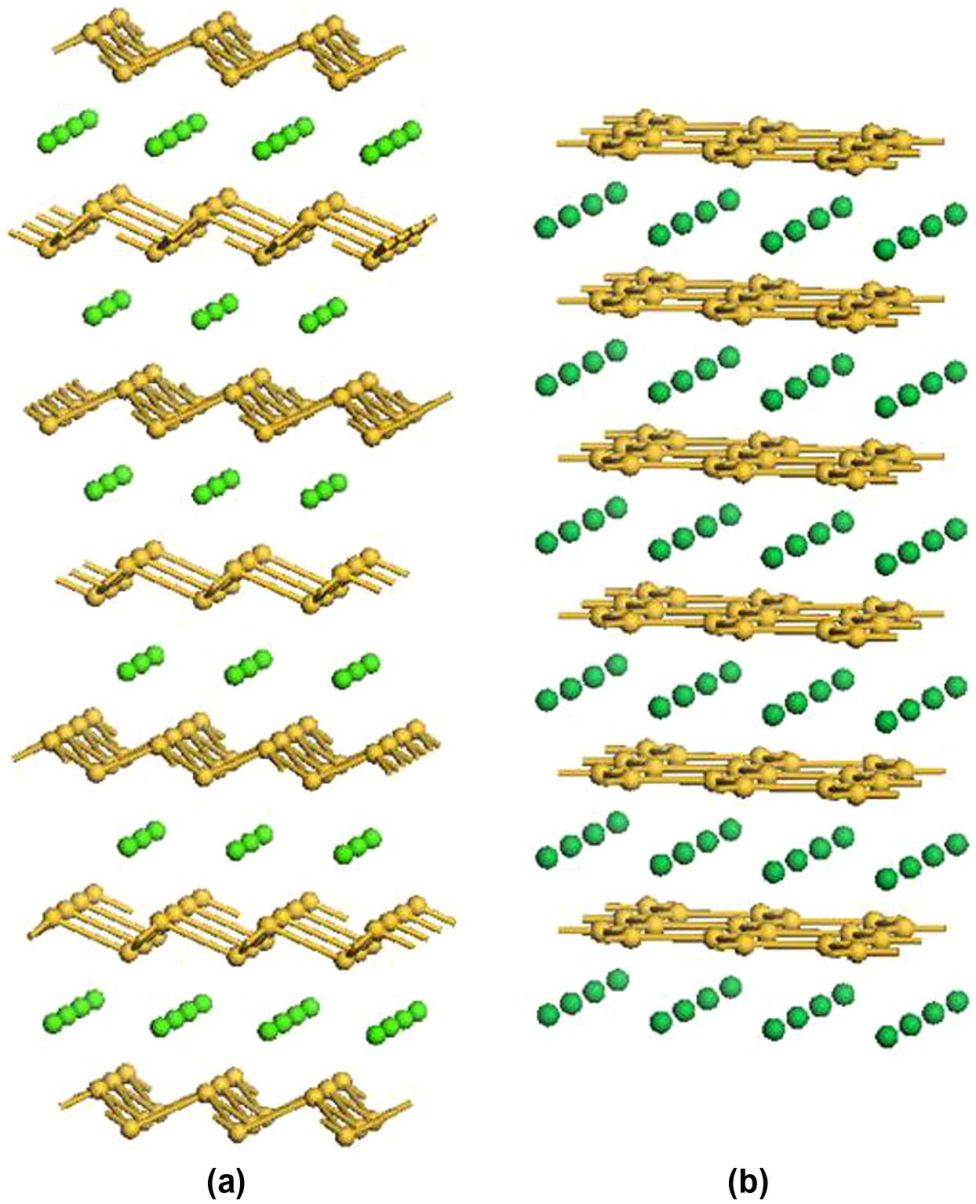
Schematic illustrations of the structures of Zintl binary silicides: (a) CaSi_2_ and (b) AlB_2_ type structure. The yellow-colored balls are silicon atoms.

Zintl binary silicides with alkali metal, alkaline-earth metal, and lanthanide elements are important precursors for silicon nanomaterials. The alkaline-earth metal and lanthanide disilicides (MSi_2_) adopt a variety of silicon sub-networks depending on the size of the metals. For example, the cubic root of the cell volume per unit formula of MSi_2_ decreases in the following order BaSi_2_ > SrSi_2_ > CaSi_2_ > α-ThSi_2_ > the AlB_2_ structures [73]. In these MSi_2_ structures, CaSi_2_ has 2D corrugated silicon sheets (Figure 8(a)), similar to the structure in black phosphorus [74]. On the other hand, the AlB_2_ structures (such as YbSi_2_, ErSi_2_, TmSi_2_, and LuSi_2_) have 2D silicon flat sheets (Figure 8(b)) like those of graphite. These disilicides can be regarded as metal-intercalated silicenes.

Although the AlB_2_ structures, in which the lanthanide elements have a high oxidation potential, cannot be oxidized with aqueous hydrochloric (HCl) acid solutions, CaSi_2_ can be oxidized with aqueous solutions of HCl. In a concentrated HCl, at room temperature, CaSi_2_ is oxidized with water to form siloxene: [75]

$${\text{n CaSi}}_{\text{2}}{\;\text{+ 2n HCl}\;\;\text{+}\;\;\text{n H}}_{\text{2}}\text{O}\to {\left[{\text{Si}}_{2}\left(\text{OH}\right)\text{H}\right]}_{\text{n}}\;\text{+}\;\;{\text{n H}}_{\text{2}}\text{.}$$

In a concentrated HCl solution at temperatures below –30 °C, the oxidation is suppressed, and a layered polysilane (SiH)_n_ structure was obtained without the evolution of hydrogen:[76,77]

$${\text{n CaSi}}_{\text{2}}\;\text{+ 2n HCl}\to {\left(\text{SiH}\right)}_{\text{2n}}{\;\text{+ n CaCl}}_{\text{2}}\text{.}$$

**Figure 9.**
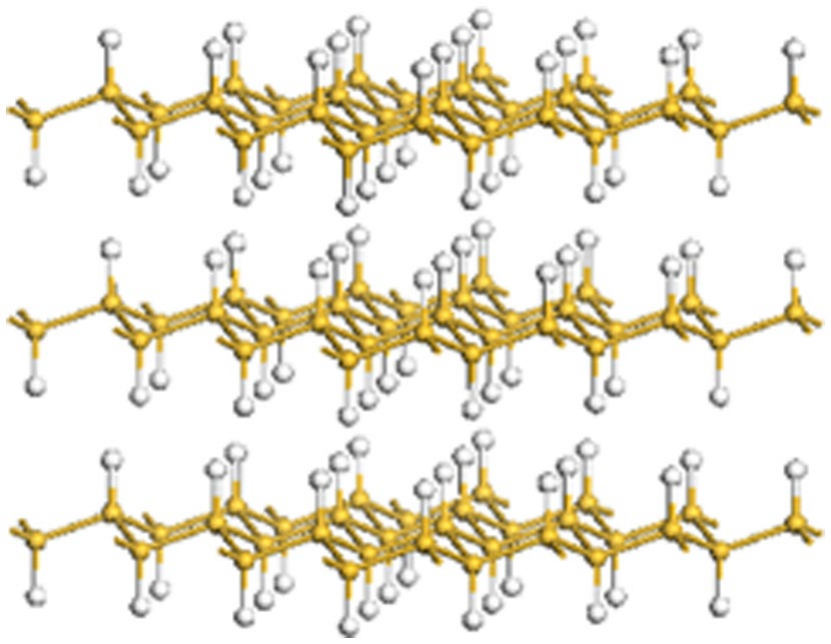
Structure of layered polysilane (SiH)_n_.

In the above reaction, the 2D silicon framework of (SiH)_n_ is maintained and all silicon atoms are capped with hydrogen atoms, as shown in Figure 9. This structure is isostructural with fluorographite (CF)_n_. Furthermore, chemically modified silicanes have been derived from these layered silicon compounds (CaSi_2_, (SiH)_n_, [Si_2_(OH)H]_n_), as described below.

### 3.2. Electrical properties of Ca-intercalated silicene and H_2_O-adapted silicane derivatives

The electronic properties of silicene in a CaSi_2_ single crystal have been analyzed by high-resolution angle-resolved photoemission spectroscopy (ARPES) [78]. In the ARPES-derived band structure, a massless Dirac cone of dispersed π-electrons at the K(H) point in the Brillouin zone was clearly observed, together with σ-band dispersions at the Γ point. Furthermore, the Dirac point was located at approximately 2 eV from the Fermi level, thereby revealing a substantial charge transfer from the Ca atoms to the silicene layers. The ARPES results indicated that the *sp* ^*2*^ bonding framework essentially maintains the CaSi_2_ structure, thereby producing a massless Dirac-cone state at the K point, despite the strongly buckled structure of the silicene layers (i.e. the graphene-like electronic structure is stably formed in this metal-intercalated multilayer silicene).

**Figure 10.**
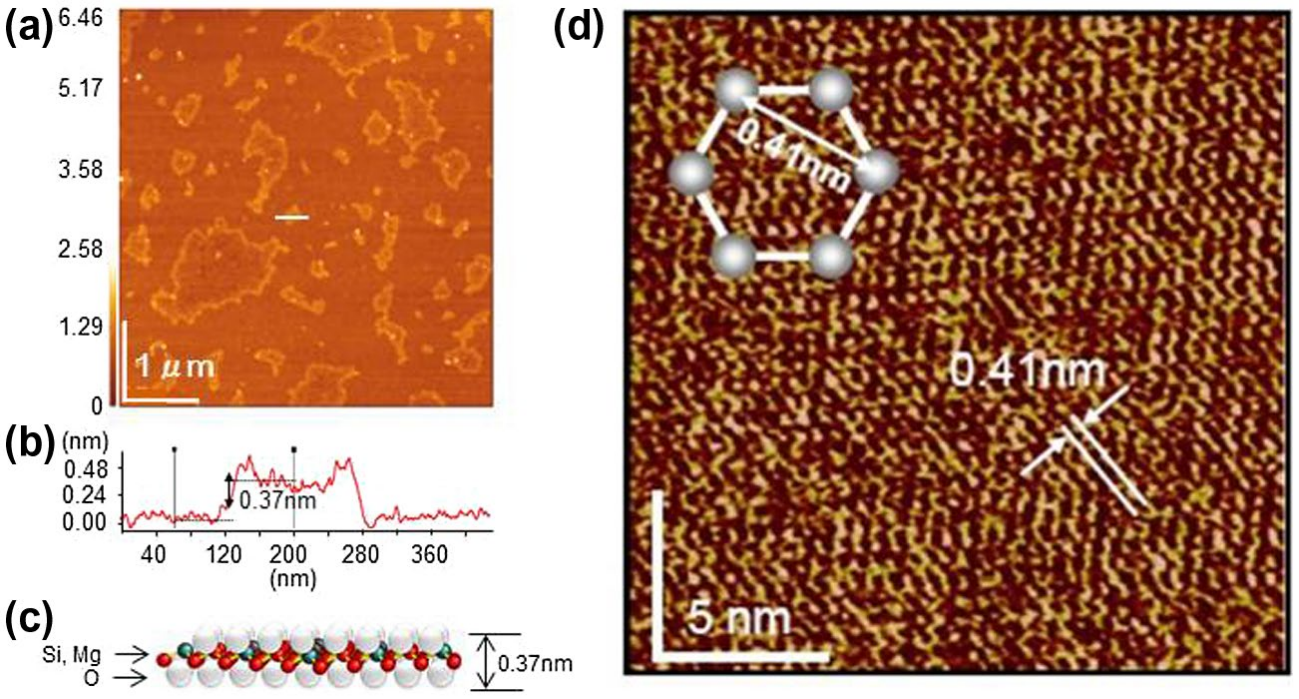
(a) AFM image of the water-adapted silicane and (b) A height profile taken along the white line. (c) Schematic illustration of the water-adapted silicane. (d) Atomically resolved AFM image. Reprinted with permission from [79]. Copyright 2006 Wiley-VCH.

In an aqueous solution of propylamine hydrochloride, Mg-substituted CaSi_2_ yielded a water-adapted silicane, which is stable against oxidation [79]. The dimensions of the silicane were determined by atomic force microscopy (AFM), and the sheets were found to be 0.37 nm thick with lateral dimensions ranging from 200 to 500 nm (Figure 10(a) and (b)). The crystallographic thickness of silicene, (without functional groups 0.16 nm) was calculated from its atomic architecture, and the difference between this value and that obtained by AFM indicates that the surface of the silicane was stabilized via capping with oxygen atoms (Figure 10(c)). As revealed by the high-resolution AFM images, the closest distance between atoms (i.e. dot-like marks in the AFM image) was 0.41 ± 0.02 nm (Figure 10(d)), which was slightly larger than the distance between Si atoms in the Si(1 1 1) plane of bulk crystalline silicon (0.38 nm). These sheets were considered to be the first examples of free-standing silicane derivatives.

### 3.3. Experimental studies of functionalized silicanes

Designing the surface chemistry of silicon nanomaterials draws on established molecular silicon chemistry as well as pioneering work related to bulk silicon surfaces. Despite some similarities, there are numerous examples where differences in the resultant materials have been reported when standard silicon chemistry methods have been applied to silicon nanomaterials. Thus, it is essential to explore the reactivity of silicanes if their full potential is to be realized. The hydrosilyl (Si–H) group on the surface of (SiH)_n_ is polarized as Si^δ+^–H^δ−^ due to the smaller electronegativity of silicon compared to that of hydrogen, and can be utilized as a reaction point for the chemical modification of silicanes. The reactivity of the Si–H group has been widely studied in the field of silicon chemistry using various methods and has been used to synthesize functionalized silicanes [80,81]. Thus, the solubility of these compounds against organic solvents is increased, allowing the materials to be exfoliated into individual sheets (and hence creating functionalized silicanes).

The Si–H groups can be derivatized by the direct reaction with amines; the reaction reportedly provides surface functionalities that are tethered through Si–N linkages [82–84]. Similarly, (SiH)_n_ can modified upon reaction with phenylmagnesium bromide to yield sheets bearing phenyl moieties (to produce Ph-silicane) or by employing widely utilized hydrosilylation reactions that are well established for other silicon nanomaterials and bulk silicon [85,86].

**Figure 11.**
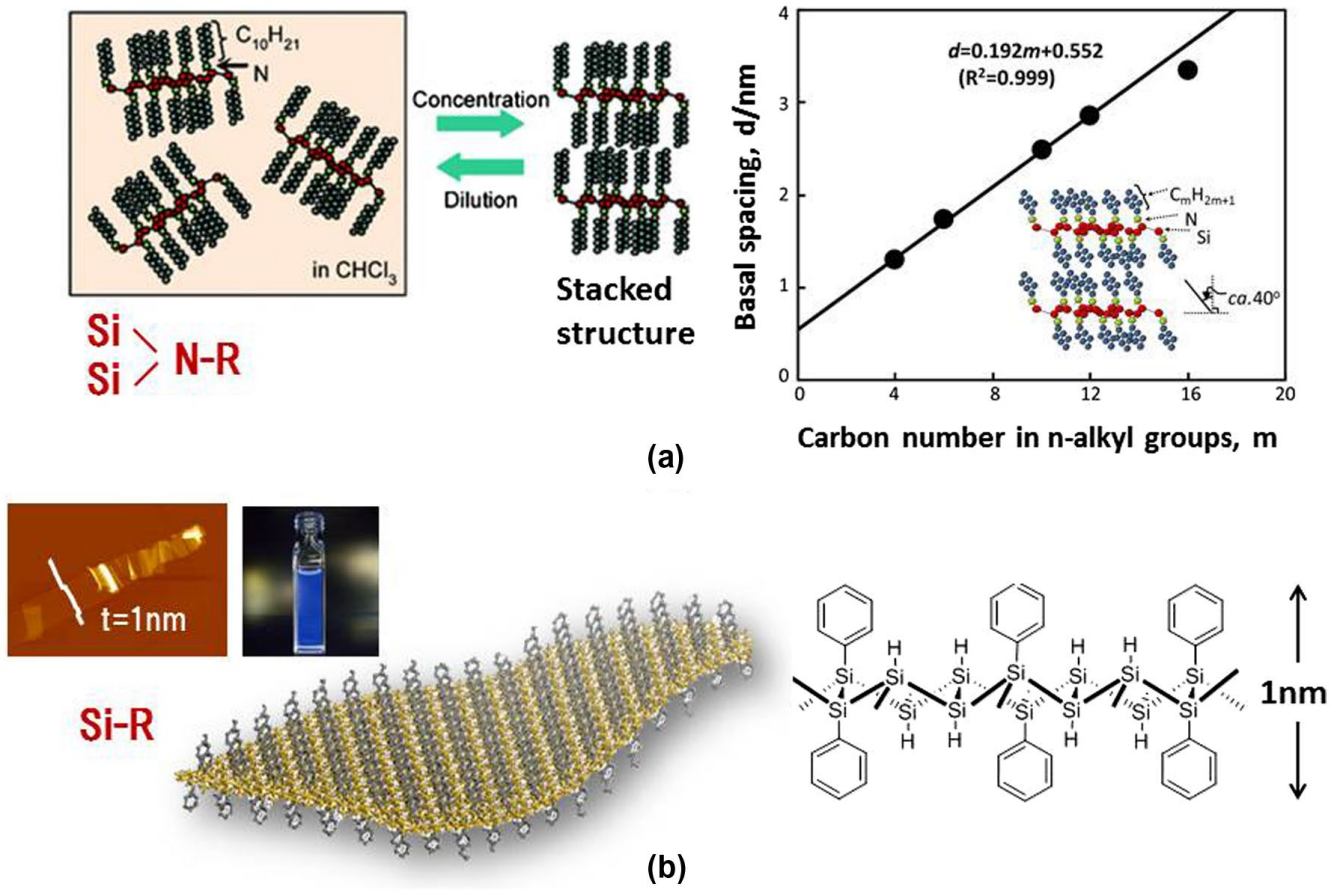
(a) Stacking properties of amino-modified silicanes. (b) AFM image, a photograph showing the PL of a Ph-silicane solution, and the schematic model of Ph-silicane.

For example, amino modification of silicane was conducted by stirring (SiH)_n_ with primary n–alkylamines, such as n–butyl–(C4), n–hexyl–(C6), n–decyl–(C10), n–dodecyl–(C12), and n–hexadecyl–(C16) amine in chloroform [82]. These materials were monodispersed in chloroform, and changed to a colloid suspension after the reaction was complete. After evaporating the solvent, very sharp (0 0 1) peaks and the appearance of many (0 0 l) peaks indicated that the amino-modified silicanes were regularly stacked parallel to the surface of the glass plate by self-assembly. The interlayer distances for the *m* = 4, 6, 10, 12, and 16 (m; carbon number) samples were calculated from the (0 0 1) reflection as *d* = 1.30, 1.72, 2.48, 2.81, and 3.35 nm, respectively. Figure 11(a) shows the proportional relationship between the d spacing of the stacked amino-modified silicanes and the m, where the slope and the intercept of the line was 0.192 and 0.552 nm, respectively. This relationship suggests that the alkyl chains included in the stacked amino-modified silicanes occur in a regular array and adopt the same conformation. The slope of 0.192 nm indicates a bilayered alkyl-chain structure at a tilt angle of ca. 40° with respect to the stacking layers.

An aryl moiety, having photoelectric characteristics, has also been attached to the silicane surface using the same synthetic method [85]. The samples obtained exhibit different optical characteristics depending on the nature of the aromatic unit; hence, the UV–Vis absorption and PL bands that were due to both the aromatic units and the silicanes, consisting of a two-dimensional silicon framework, were observed in each sample. The AFM image of Ph-silicane showed a flexible and flat plane monolayer sheet having a thickness of approximately 1.0 nm (Figure 11(b)), which is in good agreement with the thickness of the structural model of Ph-silicane. The colloidal solution of Ph-silicane in 1, 4-dioxane showed a PL emission (350 nm excitation). The position of the PL peak at 415 nm was in good agreement with the theoretical direct band gap for the calculated silicanes (3 eV). This result also supports the fact that silicanes functionalized with phenyl groups and larger aromatic molecules are expected to be useful in solar cells.

(SiH)_n_ can be also be functionalized with a variety of unsaturated functional groups using a hydrosilylation reaction. In these reactions, Lewis acids or Pt are found to efficiently catalyze the hydrosilylation reaction. Rieger’s group reported the surface modification of silicanes with a variety of unsaturated substrates, and they demonstrated the synthesis of new silicon/semiconducting polymer-based field-effect transistors [87].

### 3.4. Theoretical studies of functionalized silicane

The theoretical studies of functionalized monolayer silicane have been quite numerous and have included a number of different atoms or chemical groups that have been used to terminate the silicane. By altering the functional groups attached to the silicane, it has been shown that the electronic properties of the nanomaterial can be altered while generally retaining the structural integrity of the silicon nanosheet. Importantly, it has been demonstrated that inclusion of van der Waals forces in the calculations are necessary in a number of cases to accurately represent the structure and properties of the material.

Most theoretical studies of functionalized silicane have used density functional theory (DFT) calculations. In order to take into account the weak forces that play an important role in these materials, a variety of methods have been used, including different vdW-DF [88–90] and vdW-DF2 [91] methods as well as the DFT-D2 method of Grimme [92]. A number of hybrid functionals, such as MO6-L [93,94] and HSEH1PBE [95,96] have also been used, primarily to calculate more accurate band gaps as DFT methods are known to underestimate the size of the band gap.

In order to create different functionalized silicanes, the atoms or fragments are chemically bonded to the under-coordinated Si atoms on either one side or both sides of the silicane. In some cases, different coverages of the fragments are modeled or a combination of an organic group and H atoms are considered.

#### 3.4.1. Atom-functionalized silicane

Hydrogen attachment to silicane is the simplest termination that has been considered for functionalizing this nanomaterial. Structures with different coverages of H atoms attached to either side of the nanosheet have been modeled with the fully terminated silicane structure being called silicane. The reader can consult Refs. [97–100] for details on the studies examining this nanomaterial as well as a more recent article by Rouhi [101]. Overall, functionalization of silicane with hydrogen has been shown to widen the band gap, which is calculated to be ~2.3 eV for full surface functionalization. By varying the hydrogenation ration it was shown that the band gap of the unmodified silicane could be modified from a zero gap semiconductor to an insulator. Generally a chair-like configuration was more stable than a boat-like configuration. For the former, neighboring H atoms are alternate on both sides of the silicane sheet while for the latter, the H atoms alternate in pairs.

Termination of silicane with halogens (F, Cl, Br, I) [102–106] and alkali earth and light metals (such as Li and Mg) [107–110] has also been considered using DFT calculations. Similar to silicane, halogen-terminated silicane was calculated to have a wider band gap than the non-terminated silicane, however, the gap was not as large as for silicane. The larger electronegativity of the halogen atoms and hence more ionic nature of the Si-X bonds were suggested to weaken the Si–Si bonds and hence result in the band gap widening.

Moving to an alkali metal, the structure of silicane functionalized with Li atoms has been referred to as silicel [107]. This material is a semiconductor with a calculated band gap of 0.368 eV. The structure is stable under molecular dynamics simulations to temperatures of 1500 K at which point it starts to break apart.

Silicane functionalized with other alkali and alkali earth metals, including Li, Na, K, Be, Mg, and Ca, [108] gave stable structures at 400 K for 4 ps simulation time of molecular dynamics simulations. Further, the light alkali metals were shown to bind more strongly than the alkaline-earth metal ad atoms.

Further, by mixing or varying the atoms decorating the silicane (including a mixture of Mg and H atoms, [108] Ca atoms [109] or Na, K, Mg, and Ca atoms [110]) the functionalized material showed good storage properties for H_2_.

#### 3.4.2. Molecular-functionalized silicane

Functionalization of silicane with different molecular fragments has included a variety of organic groups such as modification of the surface with hydroxyl (OH) [102], methyl (CH_3_) [102,111], phenyl (C_5_H_5_) [112–114], phenol (C_5_H_5_OH) [115], naphthyl [116], anthracyl [116] 2-(dimethylamino)ethanol or deanol (OCH_2_CH_2_NH(CH_3_)_2_) [117], acetylene (C_2_H_2_), ethylene (C_2_H_4_), styrene (C_6_H_5_CH=CH_2_) [118], hexyl (C_6_H_13_) [111], propyl (CH_2_-CH_2_-CH_3_), amine (CH_3_CH_2_NH_2_), and ethoxy (CH_3_CH_2_O) [114] nitrophenyl diazonium [119]. A summary of these studies, along with their calculated band gaps is presented in Table 2.

**Table 2.** Calculated band gap of silicane functionalized with different molecular groups.

Chemical group	Year	Refs.	Method	Band gap (eV)	Direct/Indirect	Comments
Phenol	2016	[121]	GGA (PBE) PAW	1.88	Direct	Si_12_C_24_H_28_O_4_
			GGA vdW-DF (optB88)	1.92		
			GGA DFT-D2	1.90		
Anthracyl	2016	[116]	GGA (PBE) PAW	1.72	Direct	Si_12_C_56_H_44_
Naphthyl	2016	[116]	GGA (PBE) PAW	1.99	Direct	Si_12_C_40_H_36_
Hydroxyl	2015	[102]	vdW-DF (NC-PP)	0.7	Direct	
			MO6-L (6-31G*)	1.2	Direct	
			HSEH1PBE (6-31G*)	1.5	Direct	
Methyl	2015	[102]	vdW-DF (DZ-BS) NC-PP	1.8	Direct	
			MO6-L (6-31G*)	2.2	Direct	
			HSEH1PBE (6-31G*)	2.5	Direct	
Phenyl	2015	[114]	GGA (PBE) PAW	2.0	Indirect	Si_18_C_36_H_42_
Ethoxy	2015	[114]	GGA (PBE) PAW	1.7	Direct	Si_18_C_12_O_6_H_42_
Amine	2015	[114]	GGA (PBE) PAW	1.7	Direct	Si_18_C_12_N_6_H_48_
Propyl	2015	[114]	GGA (PBE) PAW	1.8	Indirect	Si_18_C_18_H_54_
Nitrophenyl diazonium	2015	[119]	GGA (PBE) DFT-D2 /	0.79	Indirect	NDP:Si ratio = 1:8
			HSE06, PAW	0.30	NA	NDP:Si ratio = 1:18
				0.26	NA	NDP:Si ratio = 1:32
Deanol	2014	[117]	GGA (PBE) PAW	–	–	Si_10_H_8_(OCH_2_CH_2_NH(CH_3_)_2_
Methyl	2013	[111]	GGA (PBE)	1.67	Direct	Si_8_H_4_(CH_3_)_4_
Hexyl	2013	[111]	GGA (PBE)	1.66	Direct	Si_8_H_4_(C_6_H_13_)_4_
Phenol	2013	[115]	GGA (PBE) PAW	1.88	Direct	Si_6_H_4_^(^C_6_H_4_OH)_2_, para-
Phenyl	2011	[112]	GGA (PBE) PAW	1.92	Direct	Si_6_H_4_^(^C_6_H_5_)_2_

Note: GGA stand for generalized gradient approximation, PBE for Perdew, Burke, and Ernzerhof, PAW for projector-augmented wave, and NDP for nitrophenyl diazonium.

As discussed in Section 2.3, phenyl-, hexyl-, and deanol-modified silicane have been grown experimentally and later modeled theoretically. The phenyl-modified silicane [85] has a mixture of phenyl groups as well as H atoms covalently attached to the upper and lower sides of the nanosheet. The phenyl groups are located more than 6.6 Å from each other on either side of the sheet and were shown by *ab initio* molecular dynamics simulations to be dynamic, continually rotating and tilting on the nanosheet surface at a simulation temperature of 300 K [112]. Similar to other functionalized silicane structures, this material was calculated to have a band gap of ~2 eV [112,114]. Further, the DFT calculations including van der Waals forces showed that the interaction between the sheets is very weak and they have an optimum stacking distance of ~1 nm [113], which agreed with the experimental data.

Hexyl-modified silicane [111] is composed of hexyl groups attached to both sides of the silicane sheet at a separation distance of 7.17 Å. Similar to the other functionalized silicane structures, this material was calculated to have a direct band gap of 1.65 eV which could be increased or decreased by applying simulated stress to the material. By varying the length of the alkyl chain, however, the band gap was little changed.

For deanol-modified silicane [117], used experimentally as an anode in a rocking chair type secondary battery, DFT calculations showed that the dissociation of HBF_4_, which resulted in the protonated deanol group and BF_4_ anion, was key to the variation in charge within the silicane nanomaterial.

Of the functionalized silicane structures that have not been synthesized experimentally but have been examined theoretically, the smaller fragments, such as OH [102] and CH_3_ [102,111] were shown to widen the band gap of non-modified silicane, with the gap being larger for methyl-terminated silicane.

Functionalization of silicane with acetylene, ethylene, styrene [120], propyl-, amine-, and ethoxy-groups [114], and nitrophenyl diazonium [119] has also been considered, and the products were predicted to be semiconductors. Further details of these functionalized silicanes have been reviewed previously [97].

More recently, substitution of a hydroxyl group at the ortho-, meta-, or para-positions of the phenyl-modified silicane [121] showed that the para substitution site is preferred. Van der Waals forces were shown to be necessary to model this material which formed hydrogen bonds between adjacent phenol groups on the silicane sheet. It was shown that the band gap of this material was similar to the phenyl-modified silicane, as the O atoms contributed little to the electronic states on either side of the band gap region. Hence, this suggests that the electronic properties of the nanomaterial could be retained while producing a material that is considered more hydrophilic in nature.

**Figure 12.**
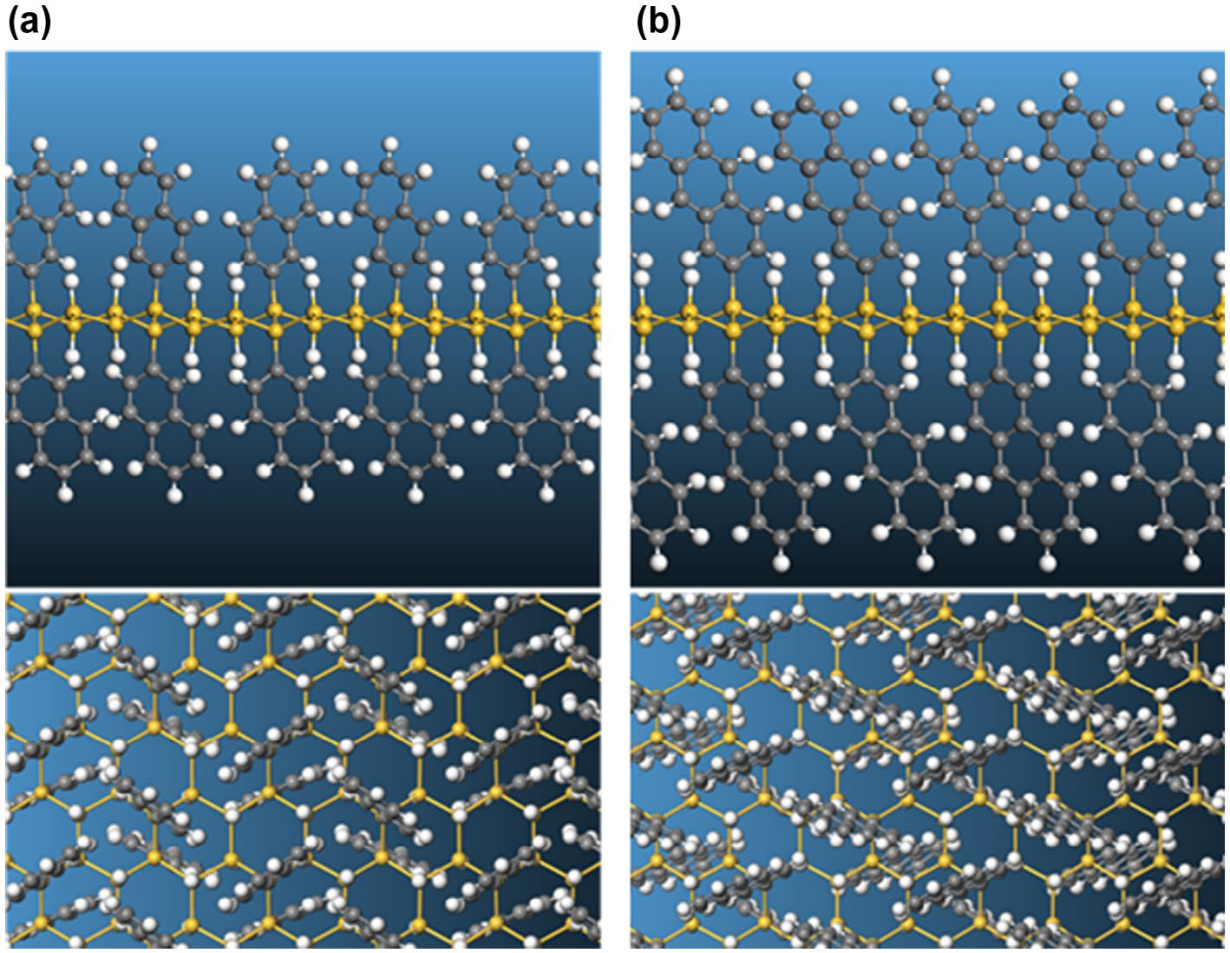
Optimized structures of (a) naphthyl and (b) anthracyl-functionalized silicane.

**Figure 13.**
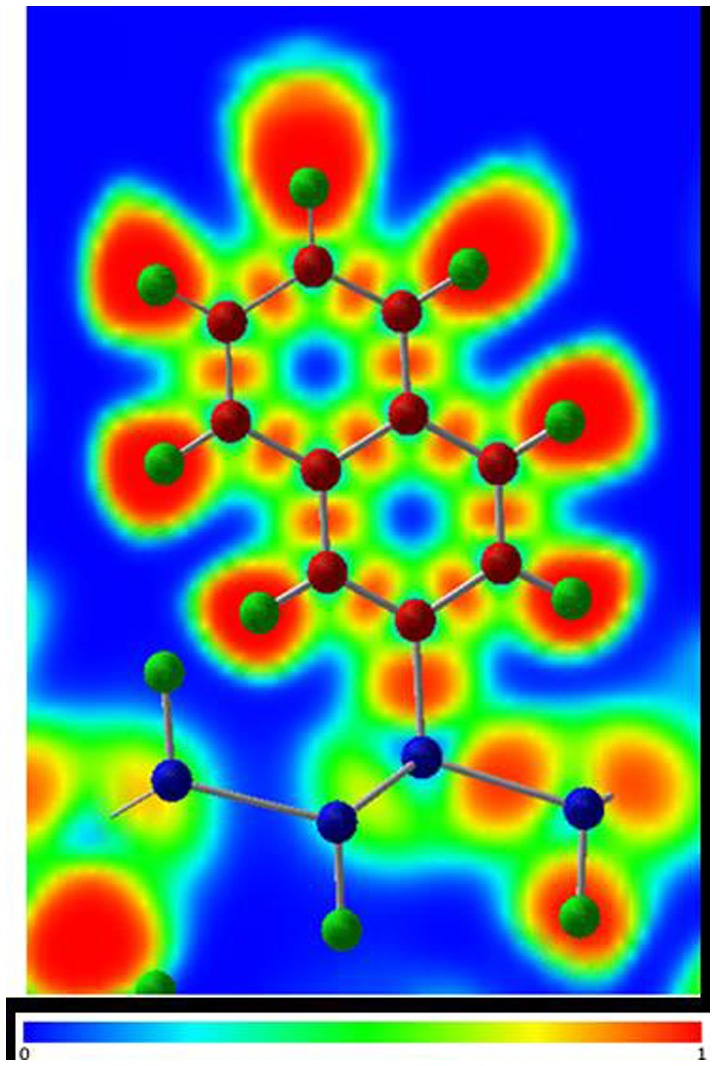
Electron localization function (ELF) plot of the naphthyl-modified silicane.

Note: The slice passes through the plane of the naphthyl group and the Si atom where it is attached.

**Figure 14.**
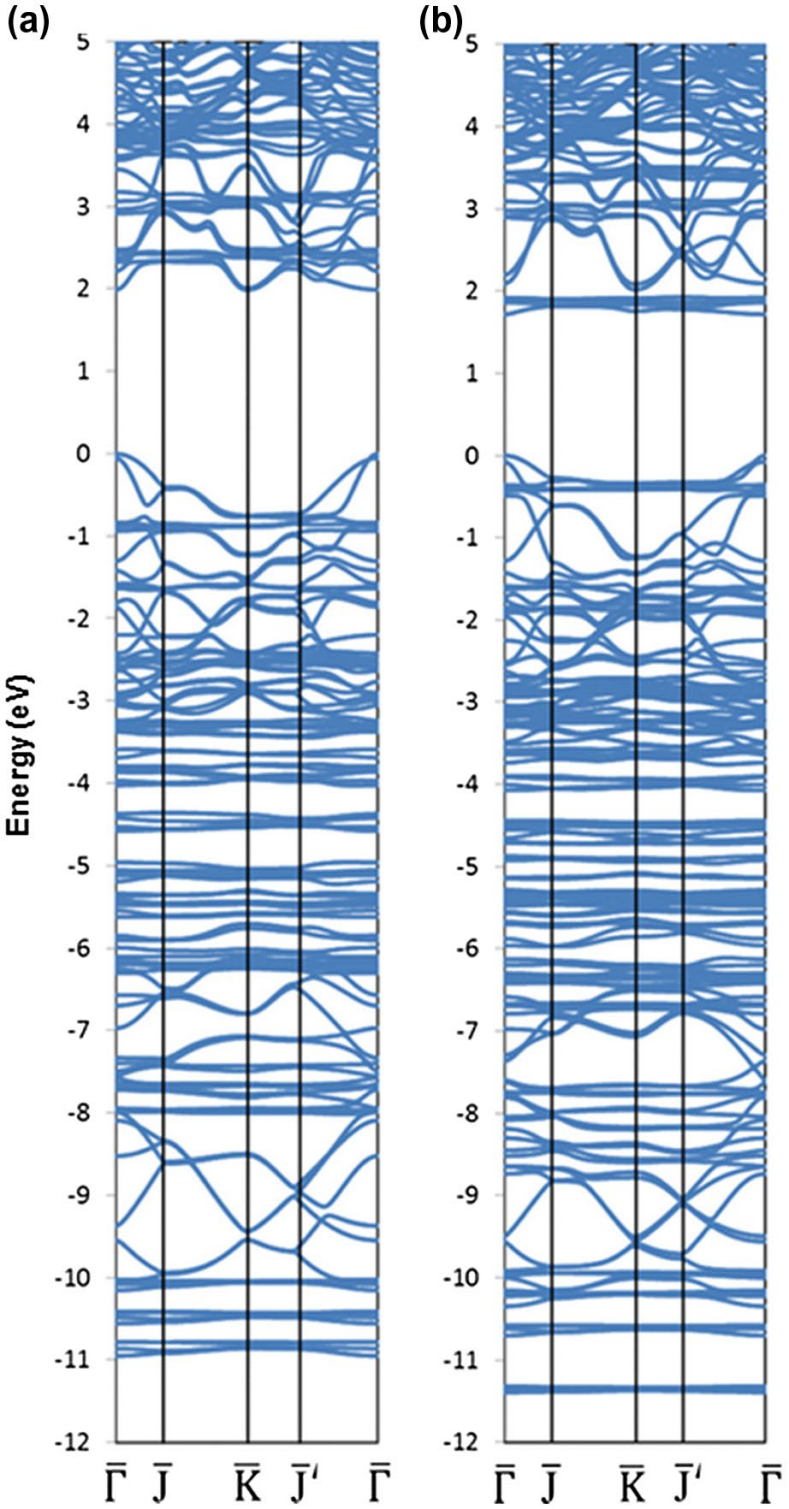
Band structure of the (a) naphthyl and (b) anthracyl-modified silicene.

Functionalization of silicane with small polycyclic aromatic hydrocarbons (PAH), namely anthracyl or naphthyl groups, using the same concentration of covalently attached organic groups and H atoms as for the phenyl-modified silicane, has also been studied using DFT calculations (Figure 12) [116]. Different nanosheet thicknesses were also considered in this work with mono-, bi-, and tri-layer silicane being modeled. For all structures, the PAH groups were shown to be covalently attached to the nanosheet, as confirmed by the electron localization function plots (Figure 13). This work showed that the band gap of the functionalized silicane was reduced in size when the length of the PAH was increased from two to three rings but both structures retained their semiconducting properties having a direct gap (Figure 14). By varying the thickness of the silicane sheet from one to three layers, the band gap could also be modified. Hence, it was predicted that the electronic properties of the material could be altered by modifying the organic group as well as the nanosheet thickness.

## 4. Bilayer silicene

Pristine monolayer silicene has been experimentally synthesized on metal substrates and its characteristics have been disclosed by both experimental [13–30] and theoretical studies [97–100]. In contrast, such attempts to synthesize and characterize multilayer silicene (MLS) have been initiated only recently [122,123]. MLS has been successfully synthesized on metal substrates in previous experiments, however, pristine MLS or free-standing MLS has not been well characterized. In this section, particular focus is given to bilayer silicene, the two-layer structure of Si. Theoretical approaches including molecular dynamics (MD) simulations and DFT calculations to characterize bilayer silicene are briefly reviewed in the first part of this section. Recent theoretical predictions and experimental synthesis of various structures of bilayer silicene will then be reviewed in the second part of the section.

### 4.1. Formation of bilayer silicene

A possible formation process of bilayer silicene was first demonstrated by Morishita et al. using MD simulations [124]. They found that MLS (bilayer and trilayer silicene) can be formed by exploiting slit pores, wherein spontaneous formation of two-dimensional crystalline structures was induced by quenching molten Si. In fact, the effect of confinement in nanopores such as porous silica and carbon nanotubes has been extensively demonstrated for a variety of materials [125–128]; for example, novel one- and two-dimensional ice structures have been found to form inside nanopores [125,126] which act as a template for these structures.

In the MD simulations by Morishita et al. [124], molten Si consisting of 512 atoms modeled by the Tersoff interatomic potential [129] was prepared in the slit nanopore with a width of 9.3 Å under zero lateral pressure (i.e. free boundary conditions in the directions parallel to the walls were imposed). The confined molten Si was equilibrated for ~ 1 ns at 2400 K, and was then quenched to 0 K at a rate of 2 × 10^10^ K/s. They found that a layered structure was formed at ~ 1700 K inside the slit nanopore.

**Figure 15.**
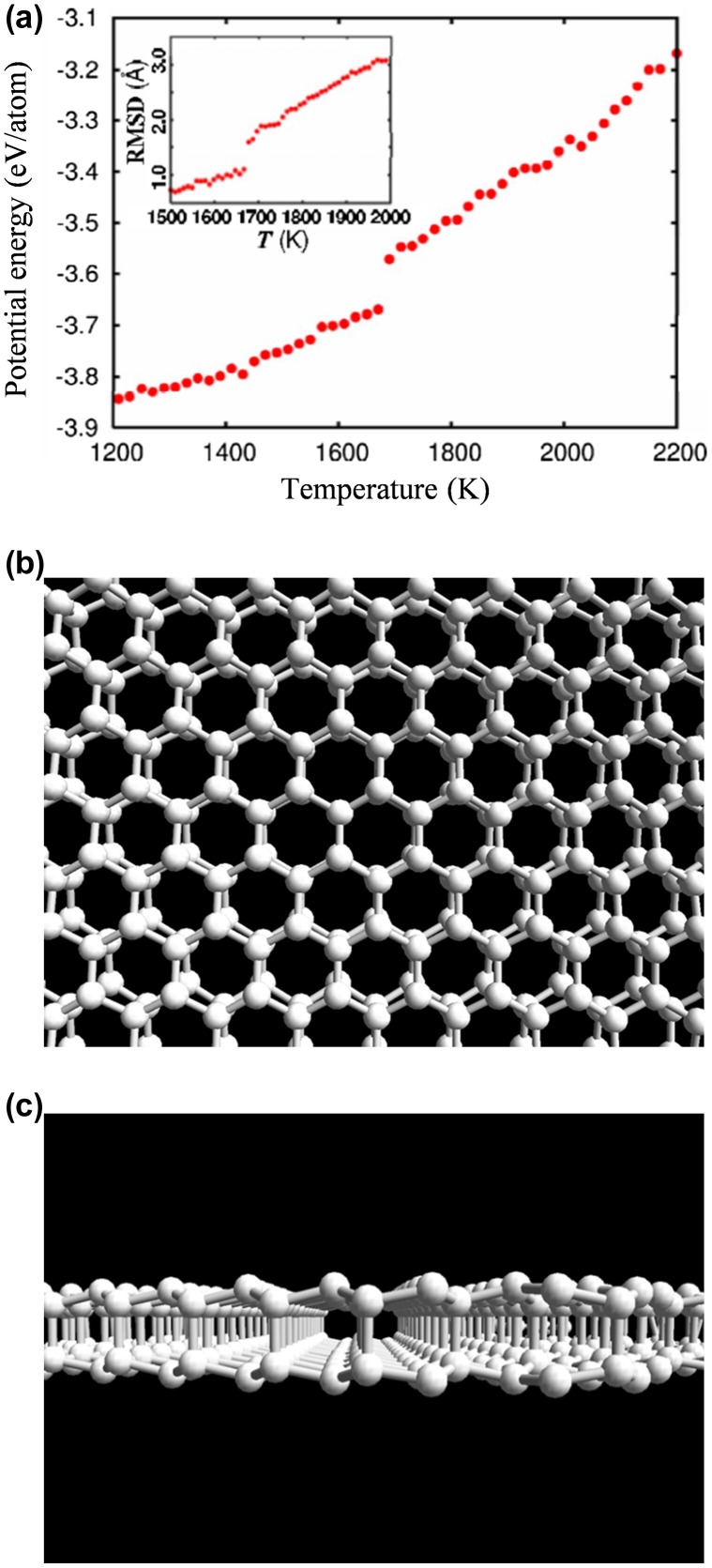
(a) Potential energy of Si confined in a slit pore as a function of temperature (Inset: 2D RMSD of Si over 10 ps). (b) and (c) Top and side views, respectively, of bilayer honeycomb silicene (BHS) obtained in the MD simulation by Morishita et al. [124]. Copyright (2008) The American Physical Society.

Figure 15(a) shows the temperature dependence of the potential energy, U, of the molten Si in the quenching process. A sharp drop at ~1700 K clearly indicates that a first-order transition from a liquid to a solid state took place. The temperature dependence of the atomic mobility was also examined. The two-dimensional root mean-square displacement (2D RMSD) parallel to the wall over a 10 ps time period (averaged over all Si atoms) is given in the inset of Figure 15(a). A sharp drop is again seen at ~1700 K, at which the 2D RMSD decreases down to less than ~1 Å indicating solidification at ~1700 K.

Figure 15(b) and (c) show the atomic configuration obtained in the quenching process (the 0 K structure). One clearly sees that two honeycomb layers are formed parallel to the walls with an AA stacking configuration. It is, thus, demonstrated that bilayer honeycomb silicene (BHS) can be synthesized by quenching molten Si confined in a slit pore.

Bai et al. [130] also performed MD simulations of molten Si confined in a slit pore using the Stillinger–Weber interatomic potential [131], as well as the Tersoff potential. They found that a bilayer silicene having a slightly different structure from that obtained in the previous MD simulation [124] was formed in the quenching process. This is actually not surprising because they employed a pore having a slit width of 7－8 Å and the lateral pressure was maintained at 50 MPa [130]. Moreover, Johnston et al. [132] discovered a variety of two-dimensional Si structures, including BHS, in their MD simulations for confined molten Si upon quenching under various lateral pressures. Their simulation results indicate that the structure consisting of the buckled honeycomb layers is not a unique two-dimensional structure of Si. In fact, recent DFT studies have demonstrated that surface reconstructions could induce structural variety of bilayer silicene, which is discussed in Section 4.3. Note that monolayer silicene has also been found to have a 2D structure without the honeycomb lattice [133].

### 4.2. Structural and electronic properties of bilayer honeycomb silicene

While the standard classical MD simulation is able to provide dynamical properties in nanoseconds or beyond, the interatomic interactions therein are often approximated by simple functions of the distance of two atoms, which may not be sufficient to accurately characterize a variety of nanoscale structures such as silicene. DFT-based calculations have, thus, been intensively performed for mono- and bilayer silicene, especially those with the honeycomb lattice, by several groups. Here, details of the structural and electronic properties of BHS estimated using DFT are discussed.

The in-plane Si–Si bond-length in the BHS structure with an AA stacking (Figure 15) was estimated to be 2.32 Å and the inter-plane bond was estimated to be 2.46–2.47 Å within the framework of DFT [134,135]. Since the bond-length in the bulk crystalline silicon (c-Si) is 2.352 Å, the in-plane Si–Si bond is slightly shorter and the inter-plane bond is longer than the bond-length in the bulk c-Si. This is not surprising because the outermost Si atoms are three-fold coordinated, thus, there exists dangling bonds on these under-coordinated (surface) Si atoms, resulting in degradation of the *sp* ^3^ hybridization and the tetrahedral bonding. The length of the in-plane and inter-plane bonds in BHS with an AB stacking has also been estimated using DFT calculations [135,136], which show the same trend as for the AA stacking structure.

**Figure 16.**
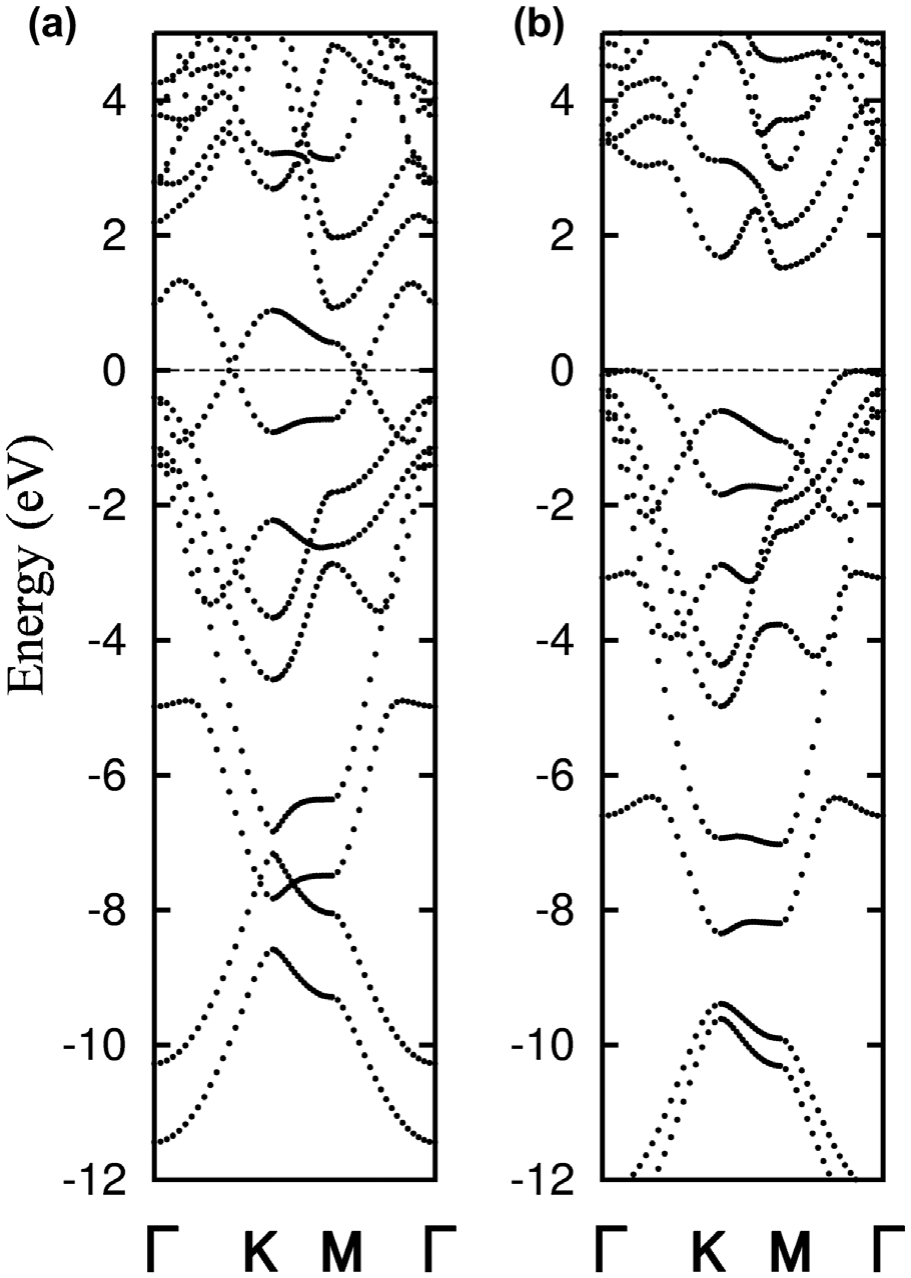
Band dispersion relations for (a) pristine BHS and (b) P-doped BHS (all the outermost Si atoms are substituted by P, thereby no dangling bond exists) [134]. Copyright (2010) The American Physical Society.

Kamal et al. [135] computed the electronic band dispersion relations for AA and AB BHS. It was found that linear dispersions, which are characteristic of the band dispersion for pristine monolayer silicene, remain in AA BHS (see also Figure 16(a)), while parabolic dispersion is instead observed in AB BHS. In fact, the same trend is also found in AA and AB graphene [137]. The linear dispersion in AA BHS is observed not at the high symmetry K point, but at the points along the Γ-K and Γ-M lines.

Morishita et al. [134] reported that the bands crossing the Fermi level basically originate from the dangling bond on the under-coordinated (outermost) Si atoms. They found that these bands can be removed by saturating the dangling bonds, e.g. by hydrogenation or substitutional doping. Figure 16(b) shows the electronic band dispersion relation for AA BHS with substitutional doping of phosphorus (P). All the outermost Si atoms of BHS are replaced with P atoms, thus, the remaining Si atoms are four-fold coordinated and no dangling bond is left. Consequently, the electron bands near the Fermi level are fully occupied and the band gap of ~1.5 eV emerges. In fact, it has been demonstrated that partial doping of P could yield acceptor levels, leading to a *p*-type semiconductor, which is in stark contrast to the P-doped c-Si being an *n*-type semiconductor. More detailed discussion can be found in Ref. [134].

### 4.3. Structural variety of bilayer silicene

While the structural and electronic properties of pristine BHS have been intensively investigated, much less is known about its stability at finite temperature. Here, we show that silicene can take a variety of bilayer structures, most of which have been obtained by performing DFT-MD simulations at 300 K.

Morishita et al. carried out a series of DFT-MD calculations to investigate the stability of BHS at 300 K [138]. They found that either the AA or AB stacking structure is transformed to a bilayer structure exhibiting novel surface reconstructions. It is well known that the cleaved Si(1 1 1) surface reconstructs at finite temperature in vacuum, creating a 7 × 7 or 2 × 1 surface structure. Such surface reconstruction is triggered by the driving force to reduce the number of dangling bonds on the surface, which also applies to BHS. Thus, BHS can be easily transformed to other bilayer structures as demonstrated in the DFT-MD simulations.

**Figure 17.**
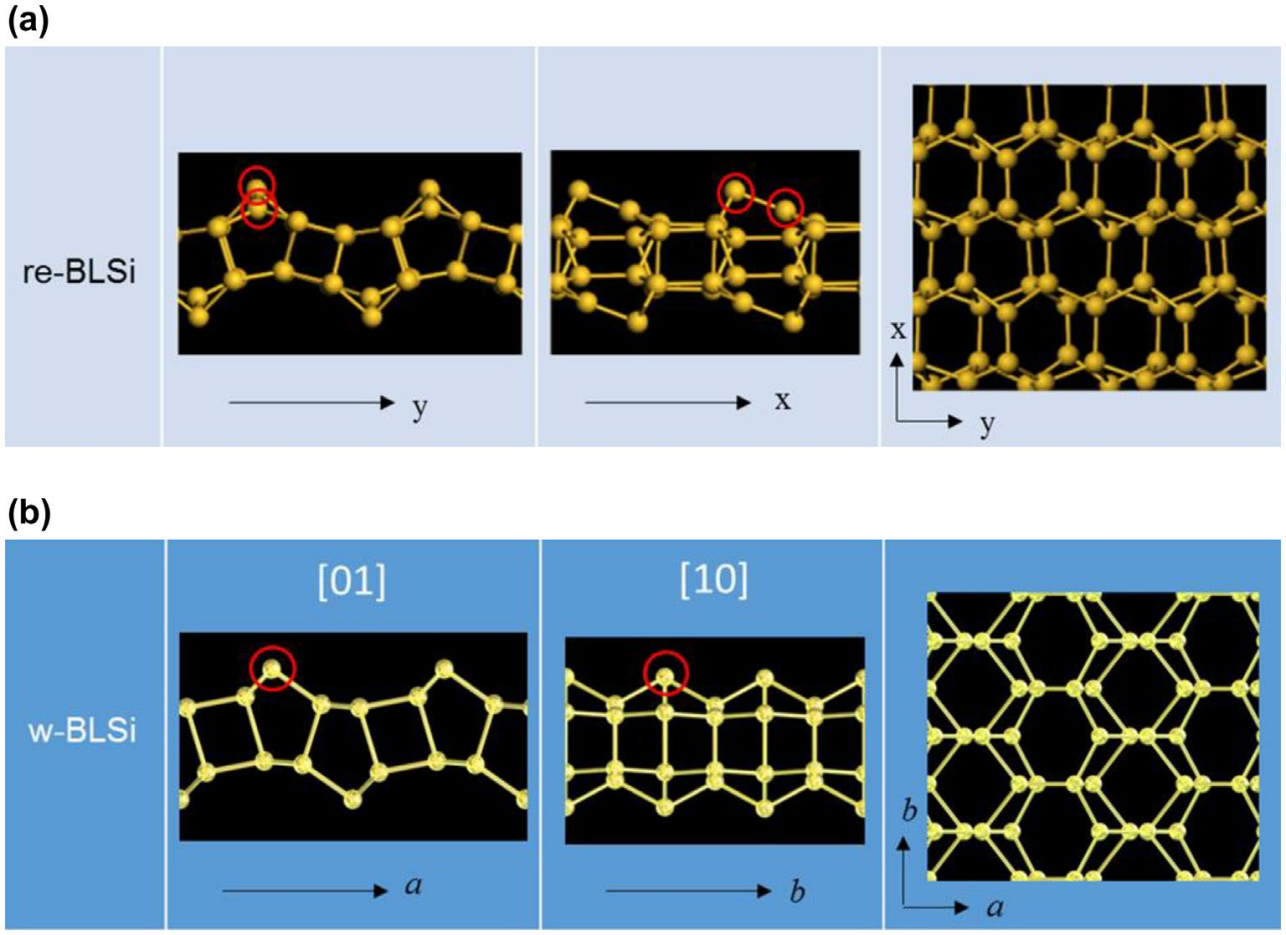
Atomic configurations for (a) re-BLS and (b) w-BLS.

Figure 17(a) shows the top and side views of the bilayer structure found by Morishita et al. [138]. The periodicity in the *x*- and *y*-directions was doubled upon reconstruction, thus, the resultant surface arrangement can be labeled as Si(1 1 1)-2 × 2 (we call this silicene ‘reconstructed bilayer silicene (re-BLS)’ hereafter). The bond-length and bond-angle in re-BLS range from 2.30 to 2.45 Å and 80° to 140°, respectively. This indicates that the tetrahedral order in re-BLS is greatly reduced upon surface reconstruction.

Several groups have also found, using DFT calculations, bilayer structures with surface reconstruction. Padilha and Pontes [139] discovered a bilayer structure having another reconstructed surfaces (which we call ‘wavy bilayer silicene (w-BLS)’ for later use [see Figure 17(b)]), and compared its band structure and scanning tunneling microscopy image with those of re-BLS. Lian and Ni [140] reported that re-BLS is stable at low pressure but it can be transformed upon compression to the structure obtained in the MD simulations by Bai et al. [130]. Sakai and Oshiyama [141] performed an extensive survey of stable structures of bilayer silicene and identified 10 (meta)stable structures including a re-BLS-like structure (with the hexagonal symmetry) which has the lowest energy among the 10 structures they identified. Their results indicate that various bilayer structures could be formed depending on the external pressure (or surface density).

Yaokawa et al. recently found [142] that BHS and w-BLS can be synthesized in a CaSi_2_F_*x*_ layered crystal (see Section 4.4). While the details on this discovery are presented in the next subsection, here, we discuss the relation between the structural stability of these bilayer silicene and charge rearrangement on their surfaces.

Their DFT calculations have shown that electrons from the Ca atoms saturate the dangling bonds on BHS, thus, stabilizing its structure inside the CaSi_2_F_*x*_ crystal. Such electrons saturating the dangling bonds, however, tend to be attracted to the F atoms when the concentration of the F increases, which results in destabilization of the honeycomb structure. BHS was, thus, transformed to w-BLS as the concentration of the F was increased in their experiment [142]. In fact, w-BLS has a higher energy than re-BLS in vacuum. The structural parameters of the silicene, however, should be consistent with those of the CaSi_2_F_*x*_ crystal, which effectively acts as an external stress. w-BLS was, thus, formed instead of re-BLS despite its higher energy under zero stress condition. In fact, a recent DFT-MD simulation has succeeded in reproducing the transformation from BHS to w-BLS by increasing the concentration of the F atoms at the interfaces between the silicene and the CaF_2_ layer in the CaSi_2_F_*x*_ crystal. More details will be reported elsewhere.

### 4.4. Synthesis of bilayer silicene

**Figure 18.**
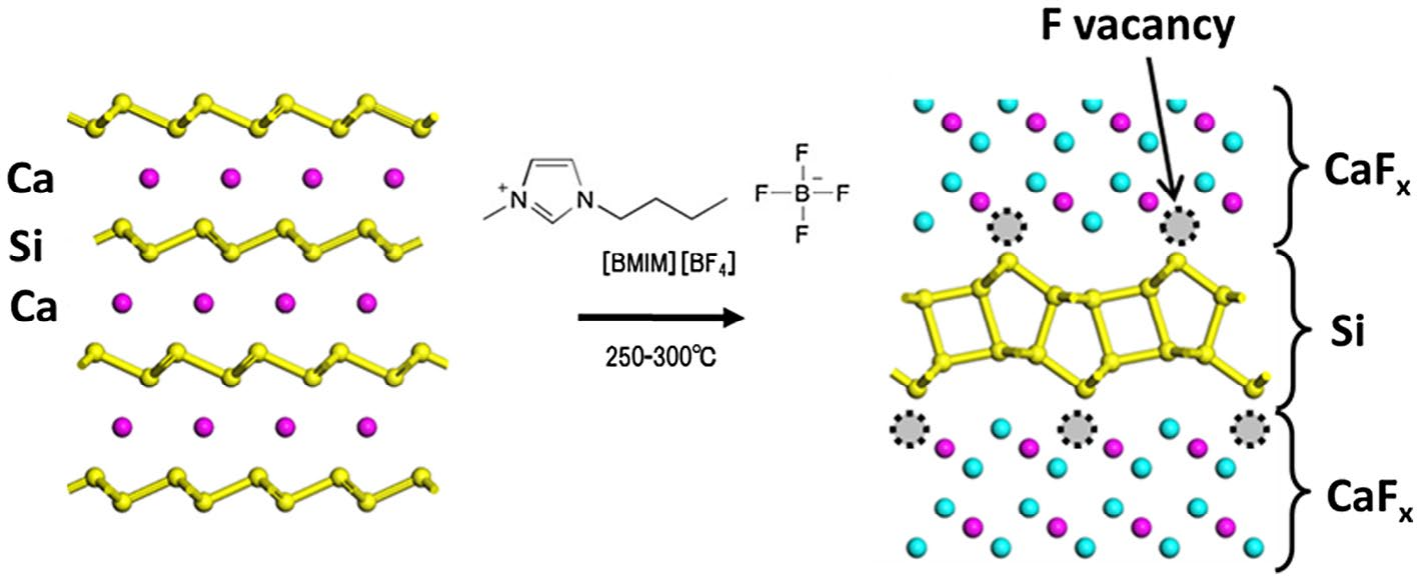
Schematic illustration of the crystal structure of CaSi_2_ and its reconstruction to w-BLSi.

**Figure 19.**
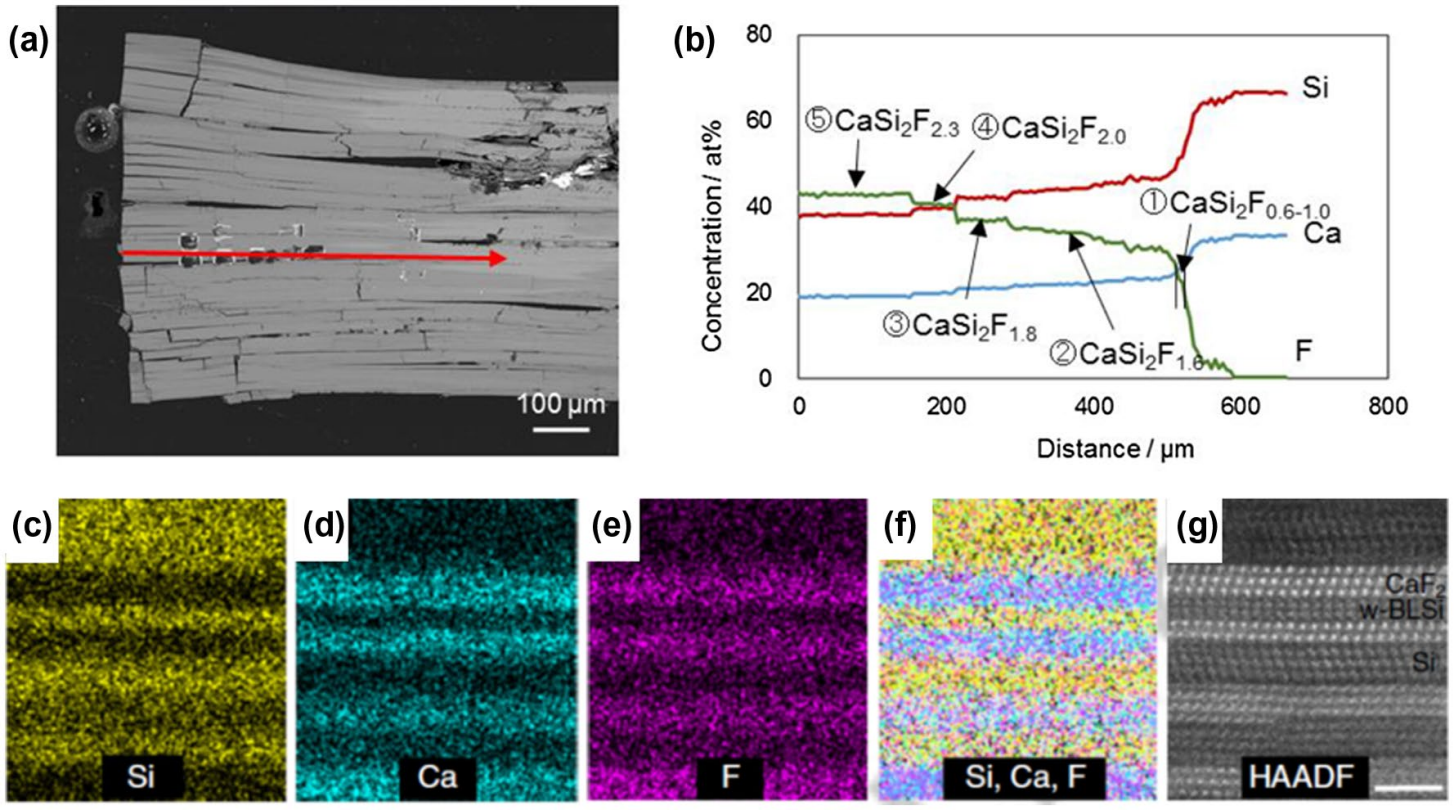
(a) Cross-sectional back-scatter scanning electron microscopy image of the crystal grain, including a CaSi_2_F_*x*_ compound. (b) Electron probe micro-analysis along the red arrow in (a). (c–f) STEM-EDX elemental mapping results of the CaSi_2_F_2_ composition region. (f) Overlay of Si, Ca, and F mapping. (g) HAADF-STEM image of the STEM-EDX elemental mapping area. Copyright (2016) by the Nature Publishing Group.

For the synthesis of bilayer silicene (BLS), CaSi_2_ was annealed in the ionic liquid 1-butyl-3-methylimidazolium tetrafluoroborate ([BMIM][BF4]) at 250–300 °C (see Figure 18) [142]. The CaSi_2_ single crystal was changed to a CaSi_2_F_*X*_ (0 ≤ *x* ≤ 2.3) compound through diffusion of F^−^, in which the local F^−^ concentration gradually decreased from the crystal edge inward (Figure 19(a) and (b)).

Elemental mapping by a combination of scanning transmission electron microscopy and energy-dispersive X-ray spectroscopy (STEM-EDX) elemental mapping identified the bright-contrast crystal domains as the CaF_2_ phase and the dark domains to be Si phases (Figure 19(c)–(f)). These planar domains were identified as trilayer CaF_2_, trilayer Si, bilayer CaF_2_, and a novel bilayer silicene (denoted as w-BLSi in Figure 19(g)) in the region of CaSi_2_F_1.8_ and CaSi_2_F_2.0_. The formation of m-BLSi is in accordance with predictions from a previous MD study by Morishita et al. The i- and m-BHS must be adjacent to a pair of CaF_2_ and CaSi_2_ crystal layers.

**Figure 20.**
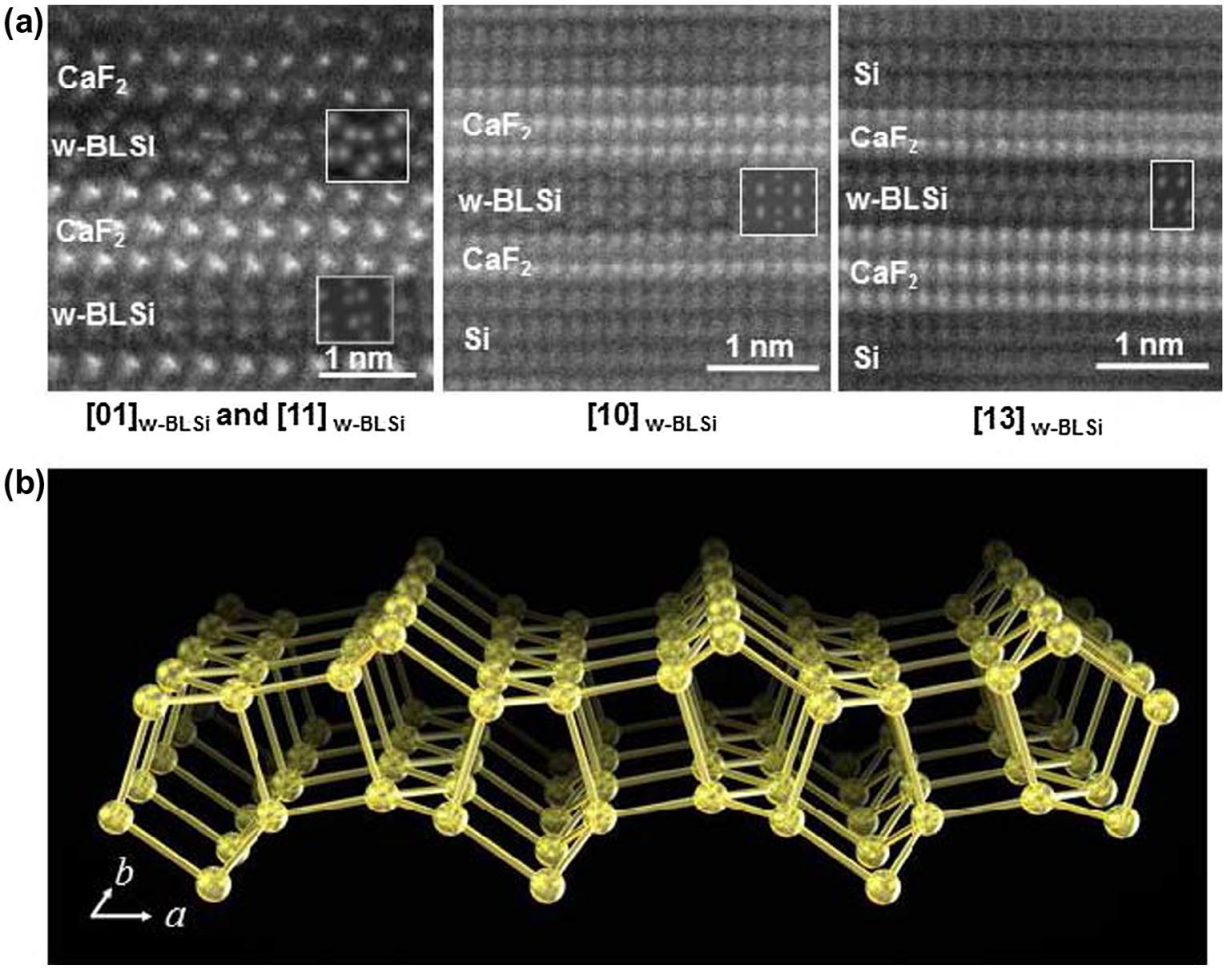
(a) HAADF-STEM images taken along three crystallographic directions and the corresponding simulation (insets) for w-BLSi. (b) Schematic of the w-BLSi atomic structure. Copyright (2016) by the Nature Publishing Group.

The 2D lattice constants of the w-BLSi were determined to be *a* = 0.661(2) nm and *b* = 0.382(3) nm from the high-resolution transmission electron microscopy images. The *a* period of w-BLSi was similar to the triple lattice spacing of d_11-2_ in CaF_2_ (0.223 nm), and the *b* period was similar to d_–110_ in CaF_2_ (0.386 nm); that is, the difference between w-BLSi and CaF_2_ (1 1 1) was less than the observation error. The atomic structure of the bilayer silicene was determined from high-angle annular dark-field STEM (HAADF-STEM) images that were taken with different incident electron beam directions (Figure 20(a)). As shown in Figure 20(b), the w-BLSi structure had a 2D translation symmetry and a wavy morphology. This structure consisted of two silicenes of alternating chair and boat conformations, which were vertically connected via four-, five-, and six-membered rings. Because w-BLSi consists of only Si atoms exhibiting tetrahedral coordination, the top atom of the five-membered silicon ring possesses under-coordinated Si atoms. Therefore, compared with those in monolayer silicene and i- (or m-) BHS, the density of under-coordinated silicon atoms in w-BLSi decreased to 25 and 50%, respectively. In almost all of the observed HAADF-STEM images, w-BLSi faced the (1 1 1) plane of CaF_2_, and the F vacancies on the CaF_2_(1 1 1) surface were recognized at special positions associated with the wavy structure of w-BLSi.

## 5. Germanane

### 5.1. Functionalized germanane

In aqueous HCl, CaSi_2_ yields partially OH-terminated SiH_*x*_(OH)_1−*x*_ due to the significantly stronger Si–O bond compared with the Si–H bond. On the other hand, the Ge–O bond is much weaker than Ge–H bond. Furthermore, any native germanium oxide or hydroxide termination is readily dissolved in aqueous HCl so as to produce pure layered polygermane (GeH)_n_. Jiang et al. have developed a one-step approach that topotactically converts CaGe_2_ into functionalized germananes [143]. Consequently, replacing the –H ligand in germanane with the more electron-donating –CH_3_ ligand will increase the observed bandgap from 1.54 to 1.67 eV. By reacting CaGe_2_ with CH_3_I, GeCH_3_ was prepared and a hexagonal unit cell was observed. In this reaction, the ${\left(\text{Ge}\right)}_{\text{n}}^{-}$ bonds to the CH_3_ group, and the iodide reacts with Ca^2+^ to form a soluble CaI_2_ species. Using this reaction as the synthetic basis, CH_3_CH_2_Ge and CH_2_=CHCH_2_Ge were prepared. The interlayer distance for –CH_2_CH_3_ and –CH_2_CH=CH_2_ were calculated from the (0 0 2) reflections, and were expected to increase by 3.5 and 6.2 Å upon replacing –H in GeH, respectively.

### 5.2. Multilayer germanenes

When reacted with ionic liquid [BMIM][BF4], germanene in Zintl-phase CaGe_2_ crystals transforms into multilayer (bilayer or trilayer) germanenes in CaGe_2_F_*x*_ (0 ≤ *x* ≤ 1.8). One of the bilayer germanenes is a new allotrope comprising four-, five-, and six-membered Ge rings, which is of the same structure as bilayer silicene [144]. The newly obtained structures of trilayer germanene are not of the cubic diamond type; however, they are described with combinations of hexagonal stacking similar to high-pressure bulk phases. Comparing the transformation of monolayer germanene into multilayer germanene in CaGe_2_F_*X*_ compounds and the above-mentioned silicene formation in CaSi_2_F_*X*_ compounds, the driving force of these transformations is the electronegativity difference between the ionic crystals. The layered structures of Ge (w-BLGe) are formed by diffusion of the more electronegative F^−^ ions into the CaGe_2_ crystal, which then causes the CaGe_2_ crystals to segregate into Ge and CaF_2_ phases, while maintaining their layered structure. However, when the F concentration reaches CaGe_2_F_1.5_, the TLGe and CaF_2_ layered structures transform into Ge and CaF_2_ crystallites. The major allotropes of TLGes sandwiched by between CaF_2_ layers did not adopt the cubic diamond structure, but adopted hexagonal structures (2H- and 4H-Ge), which are high-pressure phases because the stress derived from the misfit between the TLGe and CaF_2_ layer plays an important role in the formation of hexagonal structures. These results present the structural changes induced by fluoride diffusion into CaGe_2_ and should be relevant to structural designs for controlling misfits between interfaces. Using the tetrahedral coordination and controlling the segregation of group-IV elements, our study contributes to the design of high-performance electronic devices.

Although the transformations from monolayer to bilayer or trilayer Si or Ge are driven by the same mechanism, there are some differences between BLGe and BLSi regarding the abundance ratio of i-BL to m-BL and the lattice constants of the w-BL structure. In the case of BLSi, i-type with a partial 3C-Si atomic arrangement was preferentially formed because the misfit in the in-plane direction between CaF_2_ (0.386 nm) and 3C-Si (0.384 nm) was less than 1%, which is comparable to the observational error. Compared with the w-BL structures, the lattice constants, a and b, of w-BLGe were 2–3% larger than those of w-BLSi, and the thickness of w- BLGe (h in Figure 3(d); 0.54(3) nm) was 8% larger than that of w-BLSi (Table 3). The w-BLGe shrinks in the in-plane direction due to the compressive stress derived from the misfit between bulk Ge and CaF_2_. Consequently, the w-BLGe structure vertically expands more than w-BLSi. Therefore, the formation process of BLs and the lattice constants of the w-BL structure are influenced by the strain derived from the misfit between the contacting phases.

**Table 3.** Abundances of stacking sequences of Ca(Si, Ge)_2_ or CaF_2_/BL(Si, Ge)/CaF_2_ and lattice constants of BLSi and BLGe.

	Stacking sequences neighboring BLSi or BLGe	Si	Ge
Abundance ratio of i-BL to m-BL		i-BLSi:m-BLSi = 124:3n2fi	i-BLGe:m-BLGe = 0:43
		i-BLSi:m-BLSi = 3:0	i-BLGe:m-BLGe = 13:1
Lattice constants of the w-BL structure			
Lattice constant a		0.661(2) nm	0.681(2) nm
Lattice constant b		0.382(3) nm	0.391(4) nm
Thickness of w-BL (h)		0.501(2) nm	0.54(3) nm

## 6. Conclusions

Currently, free-standing silicene and germanene (i.e. without functional groups) have not been achieved experimentally. Nevertheless, there is a strong resemblance between the organo-modified graphene quantum dots, silicane and germanane in this text and the predicted mono-elemental free-standing 2D materials (graphene, silicene, and germanene). Therefore, knowledge of the properties of these is a good starting point for serving as a nanotechnology platform with multi-physical features for fundamental research and applications.

The theoretical and experimental studies we have reviewed, thus, far indicate that a variety of bilayer silicenes and germanenes could be synthesized depending on the conditions, such as external stress and charge distribution on the surface. These structural flexibilities mainly originate from the existence of dangling bonds on the surfaces, which place bilayer in a unique position among various nanoscale materials.

Finally, on-demand molecular design and control of the surface modification of group IV graphene analogs could be key processes used to develop electronic devices and energy storage materials in the near future. These low-dimensional materials can exist in the form of multi-layered crystals whose length and width are determined by the size of the precursor and can be greater than millimeters – suitable for exfoliation and further device processing.

## Disclosure statement

No potential conflict of interest was reported by the authors.

## Funding

This work was supported in by PRESTO; the Japan Science and Technology Agency.
